# PIEZO1‐GPX4 Axis Mediates Mechanical Stress‐Induced Vertebral Growth Plate Dysplasia via Ferroptosis Activation

**DOI:** 10.1002/advs.202502052

**Published:** 2025-07-26

**Authors:** Fei Chen, Fushuai Peng, Shuqing Chen, Yukun Du, Jianyi Li, Yuanyuan Fan, Zichen Cui, Guanghui Gu, Han Zhang, Zhensong Jiang, Guodong Wang, Xingzhi Jing, Jun Dong, Tao Li, Yongming Xi

**Affiliations:** ^1^ Department of Spinal Surgery The Affiliated Hospital of Qingdao University Qingdao Shandong 266071 China; ^2^ Department of Spine Surgery Shandong Provincial Hospital Affiliated to Shandong First Medical University Jinan Shandong 250021 China; ^3^ Shandong Public Health Clinical Center Shandong University Jinan Shandong 250013 China

**Keywords:** chondrocytes, ferroptosis, mechanical stress, PIEZO1, scoliosis

## Abstract

Current scientific consensus acknowledges mechanical stress, particularly compressive loading, as a critical contributor to the pathogenesis of vertebral growth plate disorders (VGPD), though the precise molecular mechanisms remain incompletely understood. This study establishes PIEZO1 as a mechanosensitive ion channel central to compressive stress responses. These findings demonstrate that PIEZO1 upregulation disrupts GPX4 signaling, thereby amplifying ferroptosis in vertebral growth plate chondrocytes and accelerating pathological ossification. Pharmacological inhibition of PIEZO1 effectively attenuated iron overload, mitigated oxidative stress, and suppressed mechanical stress‐induced ferroptosis cascades. Notably, both conditional PIEZO1 knockout models (Col2a1‐CreERT; Piezo1^flox/flox^) and pharmacological blockade significantly decelerate scoliosis progression. However, osteoporosis emerges as an unintended consequence of systemic PIEZO1 inhibition, likely attributable to its essential role in osteogenic differentiation. To overcome this limitation, a targeted therapeutic strategy employing micro endoscopy‐guided hydrogel‐mediated delivery of PIEZO1 inhibitors is developed, achieving spatially restricted modulation within vertebral growth plate cartilage. These results position PIEZO1 as a pivotal regulator of VGPD progression through its coordination of the PIEZO1‐GPX4‐ferroptosis axis. This work not only elucidates a novel mechanobiological pathway underlying growth plate degeneration but also introduces a precision drug delivery platform with translational potential for VGPD management. The micro‐endoscopy‐assisted hydrogel system represents a paradigm shift in localized treatment of skeletal disorders while circumventing systemic complications.

## Graphical abstract

Mechanical stress activates PIEZO1 in vertebral growth plate chondrocytes, triggering ferroptosis through direct binding and inhibition of GPX4. This axis promotes lipid peroxidation and pathological ossification, accelerating spinal deformities. Clinical and genetic evidence (Piezo1‐cKO) confirms its role in scoliosis progression. Systemic PIEZO1 inhibition attenuates deformity but induces osteoporosis; endoscopic‐guided drug loaded hydrogel delivery enables localized intervention, rescuing ferroptosis without systemic toxicity.

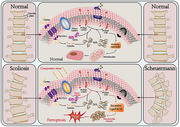

## 1 Introduction

The cartilage endplate constitutes an essential structural and functional component of spinal development. During skeletal maturation, this specialized tissue – alternatively termed the vertebral growth plate due to its homology with epiphyseal growth plates – orchestrates longitudinal vertebral growth through precisely regulated endochondral ossification. Histologically organized into resting, proliferative, and hypertrophic zones, this dynamic structure demonstrates remarkable sensitivity to biomechanical influences^[^ ^1^ ^]^ Emerging evidence underscores the critical role of mechanical loading in vertebral morphogenesis, with aberrant compressive stress representing a key etiological factor in growth plate dysfunction and subsequent spinal deformity.

Vertebral growth plate dysplasia (VGPD) encompasses clinically significant pathologies including Adolescent Idiopathic Scoliosis (AIS) and Scheuermann's disease (SD), characterized by 3D spinal deformation with respective prevalence rates of 2–4% and 0.5–8% globally.^[^ ^2^, ^3^ ^]^ Current therapeutic paradigms for these debilitating conditions range from brace‐mediated external fixation to invasive surgical interventions. While bracing demonstrates partial efficacy in deformity progression control, its inconsistent outcomes highlight the need for robust clinical validation through large‐scale randomized trials. Surgical approaches, though effective in severe cases, carry substantial risks including intraoperative trauma, postoperative complications (e.g., infections, neurological deficits, secondary deformities), and variable efficacy across severity grades. These clinical challenges underscore the imperative to elucidate the mechanobiological mechanisms underlying stress‐induced growth plate pathology, potentially enabling early detection and targeted intervention.

The unique biomechanical environment of human bipedal locomotion renders spinal growth plates particularly vulnerable to pathological loading. Compressive overload induces chondrocyte catabolism and apoptosis through mechanotransduction pathways, ultimately disrupting endochondral ossification and causing permanent deformity.^[^ ^4^ ^]^ Notably, progressive mechanical stress accumulation in AIS patients correlates strongly with scoliotic curve magnitude, particularly at concave vertebral regions.^[^ ^5^, ^6^ ^]^

Recent advances in mechanobiology have identified PIEZO1, a mechanically‐gated cation channel, as a critical mediator of cellular responses to mechanical stimuli. This evolutionarily conserved mechanosensor facilitates calcium influx in chondrocytes and other mechanoresponsive tissues, modulating diverse pathophysiological processes including programmed cell death.^[^ ^7^, ^8^, ^9^, ^10^, ^11^ ^]^ Intriguingly, spatial expression patterns of PIEZO1 in scoliotic growth plates demonstrate convex–concave asymmetry, with upregulated expression correlating with apoptosis in compressed concave regions.^[^ ^12^ ^]^

The discovery of ferroptosis (an iron‐dependent necrotic death pathway characterized by phospholipid peroxidation) has opened new avenues for understanding mechanically‐induced cartilage degeneration. Central to this process is glutathione peroxidase 4 (GPX4), whose antioxidant activity requires glutathione (GSH) to prevent membrane lipid oxidation.^[^ ^13^, ^14^, ^15^ ^]^ Mechanistic studies in articular cartilage reveal that mechanical overload upregulates PIEZO1 expression, triggering GPX4‐mediated ferroptosis through calcium‐dependent pathways. Pharmacological PIEZO1 inhibition (e.g., using GsMTx4) mitigates osteoarthritis progression in preclinical models, establishing a compelling link between mechanotransduction and ferroptosis cell death.^[^ ^16^ ^]^

Despite these advances, the potential involvement of ferroptosis in VGPD progression remains unexplored. This study employs a multidisciplinary approach combining clinical epidemiology, mechanobiological experimentation, and genetic engineering to address this knowledge gap. Using AIS as a prototypical VGPD model, we first investigate associations between systemic iron metabolism markers and scoliosis severity through retrospective clinical analysis. Complementary in vitro studies utilize a custom bioreactor system to simulate pathological loading on primary vertebral chondrocytes, enabling mechanistic interrogation of PIEZO1‐mediated ferroptosis through pharmacological modulation (Yoda1, GsMTx4) and redox pathway inhibitors.

To establish causal relationships, we generated tamoxifen‐inducible chondrocyte‐specific PIEZO1 knockout mice (Col2a1‐CreERT Piezo1^flox/flox^) using Cre‐loxP technology. This innovative model permits spatiotemporal control of PIEZO1 deletion during scoliosis progression, facilitating in vivo validation of therapeutic strategies targeting mechanotransduction pathways. Through this comprehensive methodology, our work aims to: (1) delineate the mechanochemical cascade linking compressive stress to chondrocyte ferroptosis; (2) identify novel biomarkers for early VGPD detection; (3) develop targeted interventions to halt deformity progression, potentially revolutionizing the management of spinal growth plate disorders.

## 2 Experimental Section

### 2.1 Reagents

Yoda1(HY‐18723), GsMTx4(HY‐P1410A), and Deferoxamine (DFO, HY‐B1625) were purchased from Med Chem Express (MCE, USA). *N*‐acetyl‐l‐cysteine (NAC, S1623) and Ferrostatin‐1 (Fer‐1, S7243) were purchased from Selleck (Houston, USA).

### 2.2 Clinical Study

The clinical research part was approved by the Ethics Review Committee of Shandong Hospital affiliated to Shandong first Medical University (SWYX: NO.2024‐481). Informed consent was obtained from all patients and their guardians. In this study, 105 patients with AIS who received treatment at Shandong Provincial Hospital Affiliated to Shandong First Medical University from December 2020 to February 2023 were analyzed retrospectively. This study collected full‐spine X‐ray data from each AIS patient with measurements of Cobb angles, and obtained preoperative MRI and CT data to analyze growth plate ossification and degeneration. Gender, age, and serum ferritin levels were recorded for all participants. The vertebral endplate tissue samples from hemivertebra patients mentioned in this research were obtained from tissues resected during scoliosis hemi vertebrectomy procedures at Shandong Hospital affiliated to Shandong first Medical University. Specimen collection involved no unnecessary invasive procedures beyond standard surgical requirements, and informed consent was obtained from all patients and their legal guardians.

### 2.3 Animal Model Development and Treatment

All animal procedures were approved by the Animal Care Committee of Shandong Provincial Hospital Affiliated to Shandong First Medical University (NO. NSFC2024‐0002) and the Qingdao University Laboratory Animal Management and Ethics Committee (NO. 202310C5708202502123). C57BL/6J mice were used throughout the study.

### 2.4 Experiment 1: Molecular Characterization of Vertebral Growth Plates

Thirty 3‐week‐old female C57BL/6J mice were acclimatized for 1 week. At 4 weeks of age, 15 mice were randomly selected to undergo forelimb amputation with tail resection to establish a bipedal standing model for scoliosis induction. The remaining 15 mice served as normal controls with synchronous feeding. Upright standing was encouraged by elevating food and water access. Scoliosis development was monitored via X‐ray and micro‐CT prescanning. Mice were euthanized per protocol for sample collection.

### 2.5 Experiment 2: Evaluation of PIEZO1, ROS, and Ferroptosis

Fifteen 3‐week‐old female C57BL/6J mice per group were assigned to receive: PIEZO1 agonist Yoda1 (5 µmol/kg, twice weekly); Oxidative stress inhibitor *N*‐acetylcysteine (NAC, 100 mg/kg/day); Ferroptosis inhibitor Ferrostatin‐1 (Fer‐1, 5 mg/kg, every other day). Following 1‐week acclimatization (at 4 weeks of age), interventions were administered. An additional 15 mice served as untreated controls with synchronous feeding.

### 2.6 Experiment 3: Role of PIEZO1 in Scoliosis Progression

Fifteen mice received tamoxifen‐inducible conditional PIEZO1 knockout (PIEZO1‐CKO). Fifteen mice were administered the PIEZO1 inhibitor GsMTx4 (1.5 mg/kg/week). Interventions commenced post‐scoliosis induction in the bipedal model. Fifteen scoliosis‐induced mice without intervention served as controls. The generation of PIEZO1‐CKO mice is detailed in subsequent sections.

### 2.7 PIEZO1‐CKO Mouse

The Col2a1‐CreERT mice and PIEZO1^flox/+^ mice were established and purchased from Shanghai Model Organisms Center (China). By mating PIEZO1flox/+ mice with PIEZO1^flox/+^ mice, PIEZO1^flox/flox^ mice were generated. These PIEZO1^flox/flox^ mice were then mated with Col2a1‐CreERT mice to produce Col2a1‐CreERT, PIEZO1^flox/+^ mice. Subsequent mating of Col2a1‐CreERT, PIEZO1^flox/+^ mice with Col2a1‐CreERT, PIEZO1^flox/+^ mice resulted in the production of Col2a1‐CreERT, PIEZO1^flox/flox^ mice. Female mice carrying the Col2a1‐CreERT, PIEZO1^flox/flox^ genotype were used to establish a hindlimb unloading mouse model, which mimics scoliosis. After confirming the occurrence of scoliosis through X‐ray imaging, tamoxifen (1 mg/day for 5 d; HY‐13757A, MCE, USA) was administered intraperitoneally to Col2a1‐CreER, PIEZO1^flox/flox^ mice to obtain mice with specific chondrocyte PIEZO1 knockout (PIEZO1‐CKO).

### 2.8 Genotyping

The tails of 4‐week‐old mice were harvested, and mouse tail DNA was extracted using a one‐step genotyping kit (PD101‐01, Vazyme, China), following the manufacturer's protocol. Agarose gel was prepared by mixing agarose (1.5 g) with 100 mL of Tris‐acetate‐EDTA buffer (TAE) and 6 µL of Gel Red, followed by heating. The amplified DNA was then separated using agarose gel electrophoresis. Images of the gels were captured using the Amersham Imager 680 (GE, USA). **Table** **1** provides a list of the primers (PIEZO1^flox^ and Col2a1‐CreERT) utilized for the amplification process.

**Table 1:** Primers used for Gene identification.

Target	Forward Primers,5′‐3′	Reverse Primers,5′‐3′
PIEZO1^flox/flox^ Col2a1‐CreERT	AGCAAGGCCAATGTAGTATCTGG CACTGCGGGCTCTACTTCAT	CTATTGGTGCCTAGTTGGCAGAC ACCAGCAGCACTTTTGGAAG

### 2.9 The mRNA Sequencing Experimental

Total RNA was extracted using the TRIzol reagent (Invitrogen, CA, USA). RNA purity and quantification were evaluated using the NanoDrop 2000 spectrophotometer (Thermo Scientific, USA). RNA integrity was assessed using the Agilent 2100 Bioanalyzer (Agilent Technologies, Santa Clara, CA, USA). Then the libraries were constructed using VAHTS Universal V6 RNA‐seq Library Prep Kit according to the manufacturer's instructions. The transcriptome sequencing and analysis were conducted by OE Biotech Co., Ltd. (Shanghai, China)

### 2.10 Digit Radiography Analysis

#### 2.10.1 DR Analysis

After the establishment of the bipedal upright mice model, from the second week, after intraperitoneal injection anesthesia, the mice were fixed on a special film board for X‐ray photography, and the incisors were suspended on the thin wire, and the whole plate showed a 30° angle. The scoliosis angle was measured by Cobb method, that is, the angle obtained by measuring the intersection of the horizontal extension line of the upper and lower end of scoliosis on the plain film of the anterior whole spine was the Cobb angle. A Cobb angle greater than 10° is defined as successful scoliosis induction.

#### 2.10.2 Micro‐CT Analysis

The Swiss‐made ScancoViva‐CT80 system was used in mice under isoflurane anesthesia. The scoliosis segment was first evaluated by planar reconstruction through microscopic CT scanning, and then the ossification of the long plate cartilage endplate of the upper and lower vertebral body in the scoliosis parietal region was evaluated by considering the resolution of 13 µm, 55 kVp and 145 Ma. Data processing involves the use of 3D reconstruction software.

### 2.11 Cell Isolation and Culture

The cartilage endplate was isolated from the endplate of 5‐d‐old C57/BL6 male mice. After dissection, the cartilage endplate was digested with 0.25% trypsin for 20 min and 0.25% collagenase solution for 6 h. Primary chondrocytes were collected and passaged at 80% confluence in DMEM/F12 medium (Basalmedia, Shanghai Basalmedia Technologies Co., Ltd., China) containing 10% FBS (LONSERA, Suzhou Shuangru BiologyScience & Technology Co., Ltd., China). Culture bottle and other materials were purchased from Guanzhou JetBio‐Filtration Co., Ltd. The 1st or 2nd generation chondrocytes were used in this study.

### 2.12 Cell Stress Loading

Pressure loading uses a self‐developed compressive stress loading device with independent intellectual property rights (CN202221667470.X) to carry out stress loading on cells according to demand.

Cells were seeded in 25‐mm cell culture flasks. Following administration of designated pharmacological agents or reagents, the flasks were placed within the compression chamber of a custom‐built device. The sealed chamber maintained gas composition and temperature identical to the cell culture incubator to ensure cellular viability. As static mechanical stress has been documented to induce apoptosis and alter structural characteristics, extracellular matrix composition, and gene expression,^[^ ^17^ ^]^ initial experiments quantified PIEZO1 expression across five mechanical loading gradients (0, 10, 40, 70, and 100 kPa) applied for 6 h. Subsequent experiments employed a loading intensity of 70 kPa based on these preliminary findings.

### 2.13 Chemical Stimulation Model

To determine the impact of PIEZO1 on cellular iron metabolism and ferritinophagy, 100 × 10^−6^ m ferric ammonium citrate (FAC, F5879; Sigma, USA) was used to establish an extracellular high‐iron environment. The PIEZO1 agonist Yoda1 (HY‐18723; MCE, USA) and the inhibitor GsMTx4 were introduced under this high‐iron condition. Since GsMTx4 acts as a nonspecific modulator of PIEZO1, mouse PIEZO1‐siRNA was additionally employed for target validation. Deferoxamine (DFO) was utilized to investigate the effects of iron influx blockade. Detailed experimental protocols for each intervention are presented in the respective Results sections.

### 2.14 RT‐PCR

Total RNA was extracted using TRIzol reagent (Invitrogen, USA) and reverse transcribed using the Evo M‐MLV RT Mix kit (AG 11 728, Accurate Biology, China). For RT‐qPCR, the SYBR Green PCR Master Mix (AG 11 701, Accurate Biology, China) was utilized. The relative mRNA expression levels of the target genes were determined using the 2‐ΔΔCT method, with GAPDH serving as the endogenous reference. **Table** **2** presents the complete list of all primers used.

**Table 2:** Primers used for qPCR.

Target	Forward Primers,5′‐3′	Reverse Primers,5′‐3′
PIEZO1 COL2A1 COL10A1 KEAP1 RUNX2 BMP2 ACAN SLC7A11 ACSL4 GPX4 GAPDH	CACCGAGCCCTTTCCCAACAA GGTTTGGGGAGACCATCAAT GGATGCCTCTTGTCAGTGCTAACC ACAGCAGCGTGGAGAGATATG GTATTTCAGATGATGACAC GACATCCTGAGCGAGTTCGA GGAGCAGCAGTCACATCT TGCTGGGCTGATTTTATCTTCG TGTGAGCGCATACCTGGATT CTCGCAATGAGGCAAAACCG TCTCTGCTCCTCCCTGTTCT	CCCTTCCGCACCCCAAACCAG GGTAGGTGATGTTCTGGGAG TCATAGTGCTGCTGCCTGTTGTAC GTGTGATCATCCGCCACTCA GAGGGATGAAATGCTTG CACTTGTTTCTGGCAGTTCTTC CATCAGACCAGCGGAAGT GAAAGGGCAACCATGAAGAGG CAGCCGTAGGTAAAGCAGGA GGGAAGGCCAGGATTCGTAA ATCCGTTCACACCGACCTTC

### 2.15 Western Blot Analysis

#### 2.15.1 Western Blot about Cartilage Tissue

After the scoliosis of the model mice was confirmed by X ray, the total cartilage protein of the growth plates on both sides of the crest vertebra and adjacent vertebral body was extracted. Briefly, the mice with scoliosis were killed by intraperitoneal injection of excessive pentobarbital. The growth plate cartilage of the scoliosis parietal vertebrae was retained within 1 h, the growth plate cartilage on both sides of the convex concave was separated, the samples were numbered, weighed, washed with PBS for three times, cut into pieces, and then every 30 mg of tissue was added with 1000 µL RIPA splitting buffer solution (Boston, China, AR0102) and 1% protease inhibitor, and then cracked on ice for 10 h after grinding. The lysates were collected and centrifugated at 13 000 rpm for 20 min, the supernatants were collected, and total protein concentration was measured using a BCA assay kit (Boster, China, AR0146). Next, 25 µg of protein from each sample was separated by electrophoresis using a 12% SDS‐PAGE gel(Cat No. PG212; Epizyme, Shanghai, China), before being transferred to a PVDF membrane (Millipore, United States). Membranes were then blocked with 5% BSA at room temperature for 1 h and incubated with primary antibodies anti‐PIEZO1(15939‐1‐AP, Proteintech, 1:2000), anti‐SLC7A11(26864‐1‐AP, Proteintech, 1:2000), anti‐GPX4(67763‐1‐Ig, Proteintech, 1:2000), anti‐COL2(28459‐1‐AP, Cohesion, 1:2000), anti‐KEAP1(10503‐2‐AP, Proteintech, 1:2000), anti‐ACAN(68350‐1‐Ig, Proteintech, 1:2000), anti‐RUNX2(20700‐1‐AP, Proteintech, 1:2000), anti‐BMP2(66383‐1‐Ig, Proteintech, 1:2000), anti‐COL1(14695‐1‐AP, Proteintech, 1:2000), anti‐COL10(26984‐1‐AP, Proteintech, 1:2000), and GADPH (60004‐1‐Ig, Proteintech, 1:2000). Blots were incubated in primary antibody overnight at 4 °C. After washing with TBST three times, membranes were incubated with corresponding secondary antibodies (LF102, Epizyme, 1:5000) for 1 h at room temperature. Bands were detected using a Western ECL Substrate Kit (Thermo Pierce, USA) and analyzed with the Bio‐Rad scanner's built‐in software (Bio‐Rad, Hercules, CA). Band density was quantified using ImageJ.

### 2.16 Western Blot about Cartilage Cell

Chondrocytes were seeded into 6 well plates at a density of 5 × 10^5^ cells/well and cultured until 80% confluent. After treatment, total protein was extracted. cells were washed with PBS three times and lysed with 100 µL RIPA lysis buffer (Boster, China, AR0102) supplemented with 1% proteinase inhibitor cocktail for 30 min on ice. Other operations are the same as cartilage tissue.

#### 2.16.1 Co‐Immunoprecipitation (co‐ip)

Total cellular proteins were extracted using RIPA lysis buffer supplemented with protease inhibitors. After centrifugation (14 000 × g, 15 min, 4 °C), supernatants were incubated overnight at 4 °C with 8 µg of anti‐PIEZO1 antibody (15939‐1‐AP, Proteintech,1:2000) or species‐matched IgG (negative control), followed by addition of 30 µL Protein A/G agarose beads (Santa Cruz Biotechnology, USA) for 2 h. Beads were washed three times with ice‐cold lysis buffer and once with low‐salt Tris‐HCl (50 × 10^−3^ m, pH 7.4). Immunoprecipitated complexes were eluted in 2 × Laemmli buffer (95 °C, 5 min) and separated by 10% SDS‐PAGE. Proteins were transferred to PVDF membranes, blocked with 5% nonfat milk, and probed with primary antibodies against PIEZO1 (15939‐1‐AP, Proteintech, 1:1000) and GPX4 (67763‐1‐Ig, Proteintech, 1:2000) at 4 °C overnight. HRP‐conjugated secondary antibodies (LF102, Epizyme, 1:5000) were used for chemiluminescent detection (ECL, Bio‐Rad). Input lysates (10% of total protein) and IgG controls were included to validate specificity.

#### 2.16.2 Immunohistochemistry (IHC)

After tissue slices were deparaffinized, and antigen retrieval was performed, the specimens were subsequently incubated overnight with rabbit polyclonal antibodies against anti‐PIEZO1(15939‐1‐AP, Proteintech, 1:200), anti‐COL10 (26984‐1‐AP, Proteintech, 1:200), anti‐GPX4 (67763‐1‐Ig, Proteintech, 1:200), and Purified Rabbit Monoclonal Antibody against anti‐KEAP1(21K02L70, Epizyme, 1:200). After a thorough wash, each section was incubated with biotinylated goat anti‐rabbit secondary antibody (Boster, 1:500) at room temperature for 15 min. The sections were finally colored with DAB and counterstained with hematoxylin. Images of the stained slices were analyzed using the software Image‐Pro Plus. To quantify immuno‐positive cells, at least five random fields of the cartilage were selected and the ratio of immune positive cells to total cells was calculated for each field.

#### 2.16.3 Immunofluorescence Staining (IF)

Cells were seeded into 24‐well plates at a density of 1 × 10^5^ cells mL^−1^. After treatment, cells were fixed with 4% paraformaldehyde for 15 min and permeabilized using 0.5% Triton X‐100 incubated for 20 min at room temperature. After blocking with 5% BSA for 1 h at room temperature, cells were then incubated with anti‐PIEZO1(15939‐1‐AP, Proteintech, 1:200), anti‐GPX4(67763‐1‐Ig, Proteintech,1:200), anti‐COL10(26984‐1‐AP, Proteintech, 1:200) antibodies, anti‐COL2(28459‐1‐AP, Cohesion, 1:200) overnight at 4 °C. After primary antibody incubation, cells were next incubated with Cy3‐conjugated goat anti‐rabbit secondary antibody in the dark for 1 h. After washing with PBS three times and incubation with DAPI for 10 min, images were taken using a fluorescence microscope (Evos Fl auto, Life technologies, USA).

### 2.17 HE and Safranine O Staining

Safranine O staining was conducted to assess alterations in proteoglycans, utilizing an HE staining kit (C0105, Beyotime, China) and a Safranine O staining kit (G1371, SolarBio, China), in accordance with the manufacturer's prescribed protocol.

### 2.18 Perl's Prussian Blue Stain

The iron concentration in growth plate cartilage tissue sections was assessed using the Prussian Blue Iron Stain Kit (G1428, Solarbio, China). The experimental procedure was performed as follows: Paraffin‐embedded tissue sections were first deparaffinized and rehydrated through a graded alcohol series. Subsequently, iron staining was conducted according to the manufacturer's protocols provided with the kit. The characteristic Prussian blue staining observed in the sections specifically indicated the spatial distribution and relative levels of nonchelated iron ions within the growth plate cartilage tissue.

### 2.19 Transmission Electron Microscopy

Isolated growth plate cartilage samples underwent sequential fixation for ultrastructural analysis. Briefly, tissues were first subjected to primary fixation for 1 h with Electron Microscope Fixative Solution (G1102, Servicebio, China), followed by post‐fixation for 2 h at room temperature in 1% osmium tetroxide dissolved in 0.1 mol L^−1^ phosphate‐buffered saline (pH 7.4). Subsequently, the samples were dehydrated through a graded ethanol series, infiltrated with epoxy resin, and polymerized to prepare ultrathin sections. Finally, cellular ultrastructure was examined using a transmission electron microscope (HT7700, Hitachi, Tokyo, Japan).

### 2.20 Alizarin Red Staining

The mineralization of endplate chondrocytes was analyzed by alizarin red staining.^[^ ^18^ ^]^ The chondrocytes of the vertebral growth plate were seeded in a 24‐well plate at a density of 1 × 10^5^ cells mL^−1^ and cultured to 80% fusion. Next, the osteogenic induction medium (Cyagen Biosciences, Guangzhou, China) was used instead of the medium, and the cells were cultured for another 21 d. Rinse with distilled water and fix it with paraformaldehyde at room temperature for 20 min. According to the instructions provided by the manufacturer, alizarin red (G1452, solarbio, China) was used to dye at room temperature for 15 min.

### 2.21 Alkaline Phosphatase Staining

ALP staining was used to evaluate the degree of chondrocyte mineralization.^[^ ^19^ ^]^ In this study, the activity of ALP was the highest after 7 d of osteogenic culture induction. Therefore, the chondrocytes of vertebral growth plate were inoculated into 24‐well plate at the density of 1 × 10^5^ cells per well. Once they reached 80% confluence, osteogenic differentiation medium (Cyagen Biosciences, China) was applied for 7 d. On the 7th day, according to the instructions provided by the manufacturer, the cells were stained using the ALP kit (P0321S, Beyotime, China).

### 2.22 Toluidine Blue Staining

In order to evaluate chondrogenesis, toluidine blue staining was used in our study. The chondrocytes of vertebral growth plate were inoculated into 12‐well plate at the density of 1 × 10^5^ cells per well. Once they reached 80% confluence, different concentrations of Yoda1 were applied for 7 d. Rinse with PBS and fix with formaldehyde at room temperature for 30 min. Then, according to the instructions provided by the manufacturer, toluidine blue (G2543, solarbio, China) is used to dye at room temperature.

### 2.23 Detection of Glutathione Level

Intracellular glutathione (GSH) levels were quantified using the GSH Assay Kit (ab239709, Abcam, USA). Briefly, cells or tissues were homogenized in ice‐cold 5% sulfosalicylic acid, followed by centrifugation (10 000 × g, 15 min, 4 °C) to remove precipitated proteins. Supernatants were mixed with the enzymatic reaction mix (containing glutathione reductase and DTNB) and incubated for 5 min at 25 °C. Absorbance was measured at 412 nm using a microplate reader (BioTek Synergy H1), with GSH concentrations calculated against a standard curve (0–50 × 10^−5^ m) and normalized to total protein content determined by BCA assay.

### 2.24 Detection of Lipid Peroxidation Level

Malondialdehyde (MDA), a lipid peroxidation byproduct, was measured using the Lipid Peroxidation (MDA) Assay Kit (ab118970, Abcam, USA). Tissue homogenates or cell lysates were reacted with thiobarbituric acid (TBA) at 95 °C for 60 min. After cooling, fluorescence intensity was recorded at excitation/emission wavelengths of 532 nm using a SpectraMax M5 plate reader (Molecular Devices). MDA concentrations were determined from a tetramethoxypropane (TMP) standard curve (0–20 × 10^−9^ m) and expressed as nmol MDA per mg protein. All assays were performed in triplicate, and samples were protected from light to prevent oxidation artifacts.

### 2.25 Detection of Intracellular ROS

Chondrocytes were inoculated in 24‐well plates at the density of 3 × 10^5^ cells mL^−1^, and then treated with different concentrations of Yoda1 for 24 h. Next, the cells were washed with PBS and incubated with 10 × 10^−6^ m DCFH‐DA (HY‐D0940, MCE, USA) fluorescent probe at 37 °C for 20 min in the dark. The cells were washed with PBS and observed by fluorescence microscope (Evosflauto, life technologies, USA).

In order to quantify the intracellular ROS level, the cells were collected and resuscitated in serum‐free DMEM medium. Finally, the cells were further washed in serum‐free DMEM medium with 10 × 10^−6^ m DCFH‐DA(HY‐D0940, MCE, USA) at 37 °C for 20 min, and then the average fluorescence intensity was evaluated by FACSCalibur flow cytometry (BD Biosciences, Franklin Lakes, NJ).

### 2.26 Annexin V‐FITC/PI Staining

After treatment, the cells were washed with PBS twice and stained in the dark for 20 min with Annexin V‐FITC/PI apoptosis detection kit (MA0220, Meilunbio, Dalian, China) at room temperature. Next, the cells were washed with serum‐free DMEM/F12 medium and the average fluorescence intensity was evaluated by FACSCalibur flow cytometry. Annexin V+/PI‐ cells are considered to be early apoptotic cells, while Annexin V+/PI+ cells are considered to be late apoptotic cells.

### 2.27 Molecular Docking Simulation

The protein structure of PIEZO1 (PDB ID:8IMZ) and GPX4 (PDB ID:5L71) were predicted by the AlphaFold Protein Structure Database and using GRAMM Web Server for protein docking. Next, PIEZO1 was considered as receptor while GPX4 was defined as ligand. The docking results were obtained with the default parameters and the lowest‐energy docking pose was visualized using PyMol2.3.0 software. Docking energy was calculated using Hdock Server. After removing water molecules and original ligands by Pymol 2.3.0, the targets proteins were uploaded into Autodock Tools 1.5.6 for adding hydrogen, calculating charge, assigning charge, and specifying atom type, while ligand was hydrogenated, charges calculated, charges assigned, and rotatable bonds set. Next, Grid Box size and genetic algorithm were defined, whereas simulating molecular docking and visualizing the docking results.

### 2.28 FerroOrange Fe^2+^ Staining

Intracellular Fe^2+^ was detected by FerroOrange kit (F374, Dojindo, Japan). According to the instructions, 10 μ mol L^−1^ of FerroOrange was added to the cell culture medium and incubated at 37 °C and 5% CO_2_ for 60 min. The image was taken under a fluorescence microscope.

### 2.29 Iron Assay for Iron Content

The mitochondrial Fe^2+^ level was determined via the Iron Assay Kit (ab83366, Abcam, USA). Cells were rinsed with PBS and homogenized in the iron assay buffer. The supernatant was gathered, and an iron reductant was incorporated. Subsequent to mixing and incubation, the iron probe was introduced to the samples and incubated for 1 h. Ultimately, the Fe^2+^ content was measured at an OD value of 590 nm employing a microplate reader.

### 2.30 Synthesis of GsMTx4 Drug‐Loaded Hydrogel

2g chitosan powder was weighed and dissolved in 0.1 mol dilute hydrochloric acid. The powder of β‐sodium glycerophosphate (β‐GP), 5.6 g, was dissolved in 10 mL^−1^ ultrapure water under magnetic stirring. 56% β‐GP solution was obtained, sterilized with a filter membrane (22 µm) and stored in a refrigerator at 4 °C for further experiments. 10 mg GsMTx4 was taken and dissolved in 5 mL ultrapure water under magnetic stirring. The 1 mL 56% β‐GP solution was added to the 9 mL chitosan solution and stirred slowly. After continuous stirring, 0.2% GsMTx4 solution of 5 mL was added to 2% chitosan solution of 5 mL.

### 2.31 Characterization of Drug Loading in Hydrogel

After freeze‐drying of drug‐loaded chitosan hydrogel, the sample was fixed on the sample platform. The samples were gilded by sputtering coating machine, and their morphologies were observed by scanning electron microscope (SEM).

### 2.32 The Temperature Sensitivity Study

According to the former study, a total of 3 mL TICHC was placed into sample tubes. Then, the tubes were placed in 37 °C incubators to analyze temperature‐sensitive characteristics of the hydrogel using the tube inversion method.

### 2.33 The Biocompatibility and Degradability Analysis

Cytotoxicity assessment of the drug‐loaded hydrogel TIGCH was performed using Hoechst 33 342 (C1029; Beyotime, China). Following the manufacturer's protocol, cells were washed and incubated with 1 × working solution covering the samples at room temperature for 3–5 min. After aspiration of the staining solution, samples were washed 2–3 times with PBS. Nuclear morphology was examined under a fluorescence microscope.

### 2.34 Study on the Drug Release

For drug release analysis, 0.5 mL of TIGCH was placed in an Eppendorf tube and incubated at 37 °C for 30 min to achieve gelation. The formed hydrogel was incubated with 1 mL phosphate‐buffered saline (PBS, pH 7.4) at 37 °C under continuous shaking at 50 rpm. At predetermined intervals (1, 3, 5, 7 d), 0.5 mL aliquots were withdrawn and replaced with an equal volume of fresh PBS. Drug concentration was quantified by ultraviolet spectrophotometry. Measurements were performed in triplicate, with data expressed as mean±SD. Release profiles were subsequently plotted using Prism 10.0 (GraphPad Software, USA).

### 2.35 Study on the Degradation of TIGHC

TIGCH hydrogels were prepared according to the protocol and formed at the bottom of sealed vials. The vials were maintained at 37 °C throughout the experiment. At predetermined time intervals (1, 3, 5, 7, 14, 21, 28 d), degradation solutions were carefully aspirated using pipettes, and the residual hydrogel mass loss was recorded. Measurements were performed in triplicate with data expressed as mean±SD. Degradation profiles were subsequently plotted using Prism 10.0 (GraphPad Software, USA).

### 2.36 Study on the Injectability of TICHC

The liquid samples of 3 mL‐loaded chitosan hydrogel were extracted with 5 mL syringe. The hydrogel was injected with appropriate strength to observe whether the injection was unobstructed.

### 2.37 Statistical Analysis

All data are presented as the mean±SD of at least three independent experiments. Data were preprocessed by normalizing control conditions. Differences among three or more group means were analyzed using one‐way ANOVA followed by Dunnett's post hoc tests. The independent‐sample *t*‐test was used to assess the difference between the two group means. All statistical calculations were performed using *p* < 0.05 (two‐tailed) was considered statistically significant for all tests. Statistical analyses were performed using GraphPad Prism 10.0 (GraphPad Software, USA) *p <0.05, **p <0.01, ***p <0.001, and *p ≥0.05* means not significant (ns).

## 3 Result

### 3.1 The Oxidative Stress and Ferroptosis in the Growth Plate Cartilage could be the Risk Factors for the Advancement of AIS

**Figure 1:**
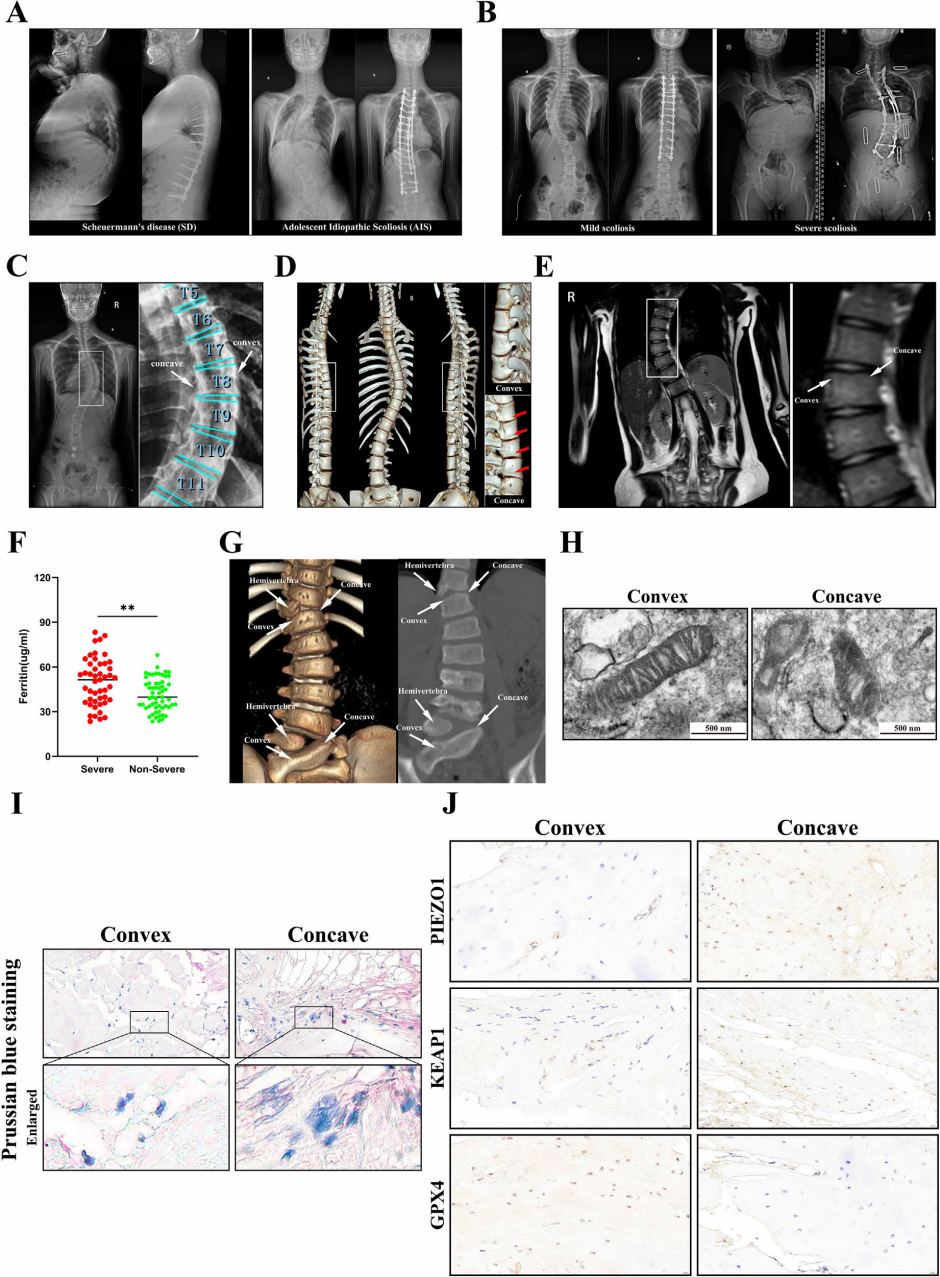
The oxidative stress and ferroptosis in the growth plate cartilage could potentially be the risk factors for the advancement of AIS. A) Typical X‐ray findings of SD patients and AIS patients show that SD manifests as abnormal spinal kyphosis, while AIS presents as spinal scoliosis, with both conditions exhibiting significant wedge‐shaped deformation of the vertebral bodies. B) Comparative analysis of preoperative and postoperative X‐ray examinations in patients with severe versus mild scoliosis revealed that severe cases exhibited less significant correction of spinal deformities compared to mild cases. C) In full‐spine X‐rays of AIS patients, asymmetric changes in intervertebral disc spaces can be observed between the convex and concave sides, with narrower disc spaces on the concave side accompanied by wedge‐shaped deformation of vertebral bodies. D) CT reconstruction of the full spine reveals wedge‐shaped vertebral deformities and asymmetric intervertebral disc changes, while also demonstrating abnormal osteophyte formation at the growth plates on the concave side (red arrow), indicating pathological ossification of vertebral growth plate cartilage. E) Coronal magnetic resonance imaging of the full spine shows that the intervertebral discs move towards the concave side, and the vertebral body signals on the concave side are lower, appearing dark, suggesting a higher degree of ossification. F) Comparison of serum ferritin levels between the severe scoliosis group (*n* = 47) and nonsevere scoliosis group (*n* = 58) revealed significantly elevated ferritin in the severe group (*P* < 0.01), indicating iron accumulation. G) Obtain the convex and concave endplate tissues from patients who have undergone hemivertebra resection. H) Transmission electron microscopy revealed that the endplate chondrocytes on the concave side of the scoliosis showed typical manifestations of mitochondrial damage. I) Prussian blue staining revealed that the concave endplate had a higher degree of staining than the convex endplate, suggesting iron accumulation. J) IHC analysis of the expression of PIEZO1, GPX4, and KEAP1 in the concave and convex endplates. All statistical data are presented as mean ± SD, *n* ≥ 3. ns (not significant), *P < 0.05, **P < 0.01, ***P < 0.001. Statistical analyses were performed using GraphPad Prism software, with one‐way ANOVA followed by Dunnett's post hoc tests and the independent‐sample *t*‐test.

In VGPD, spinal deformities manifested as abnormal kyphosis, while AIS presented with scoliosis. Both conditions exhibited vertebral wedging at affected segments (**Figure** **1**A). Orthopedic surgery represented the most effective intervention for VGPD. Mild cases achieved satisfactory deformity correction postoperatively, whereas severe cases demonstrated substantial residual deformity due to technical challenges, yielding suboptimal correction outcomes (Figure 1B). Radiographic evaluation of the scoliotic region (T5‐T11) in AIS patients revealed asymmetric disc spaces with narrowing on the concave side, accompanied by vertebral wedging, suggesting growth plate degeneration in the concavity (Figure 1C). 3D CT reconstruction further identified abnormal osteophyte formation at the concave growth plates, indicative of aberrant chondral ossification (Figure 1D). Coronal whole‐spine MRI sequences showed hypointense signals in concave vertebral bodies, reflecting advanced ossification (Figure 1E). Collectively, these findings demonstrate significant chondral degeneration and pathological ossification at the concave growth plates in AIS, driving progressive vertebral wedging.

Based on clinical diagnostic criteria defining severe scoliosis as a Cobb angle >90°,^[^ ^20^, ^21^ ^]^ patients were stratified into mild and severe cohorts (**Table** **3**). Comparative analysis of serum ferritin levels revealed significantly elevated concentrations in the severe scoliosis group versus the mild group (Figure 1F), indicating potential systemic iron overload in severe cases. This finding carries clinical relevance as iron overload is established to trigger ferroptosis‐mediated degeneration of cartilaginous endplates, thereby accelerating pathological ossification.^[^ ^22^ ^]^ Furthermore, epidemiological studies consistently report higher scoliosis incidence among individuals with iron overload compared to the general population.^[^ ^23^, ^24^, ^25^, ^26^ ^]^

**Table 3:** Demographic and clinical characteristics of the two groups defined by severity of scoliosis.

Variable	Severe group (*n* = 47)	Non‐Severe group (*n* = 58)	*P*
Age [y]	16.40±5.28	16.89±4.59	0.6132^b)^
Gender Male Female	12 35	15 43	0.9693^c)^
Ferritin [ng mL^−1^]	50.05±15.81	41.37±10.93	0.0012^a)^, ^b)^

^a)^ Statistically significant (*P* < 0.01);

^b)^ Independent *t*‐test;

^c)^ Chi‐square test.

Conventional scoliosis correction surgery employs posterior pedicle screw fixation without anterior vertebral intervention, consequently precluding intraoperative growth plate specimen collection. However, when scoliosis is accompanied by hemivertebral deformity, surgical excision of the anomalous vertebra enables harvesting of adjacent growth plate samples (Figure 1G). Transmission electron microscopy (TEM) revealed more pronounced oxidative damage and ferroptosis in concave endplate chondrocytes compared to convex regions, characterized by mitochondrial shrinkage, disrupted cristae, and increased membrane density (Figure 1H). Prussian blue staining demonstrated stronger iron deposition in concave growth plates than convex counterparts (Figure 1I). Immunohistochemical analysis showed elevated PIEZO1 expression, upregulated KEAP1 (a pro‐oxidative stress protein), and downregulated GPX4 (a peroxide scavenger) in concave regions, indicating heightened oxidative stress (Figure 1J). Collectively, these findings implicate growth plate chondrocyte oxidative stress and ferroptosis as contributing pathological factors in adolescent idiopathic scoliosis (AIS) progression.

### 3.2 Higher Degree of Ferroptosis and Cartilage Degeneration and Ossification in the Concave Vertebral Growth Plate of Scoliosis

**Figure 2:**
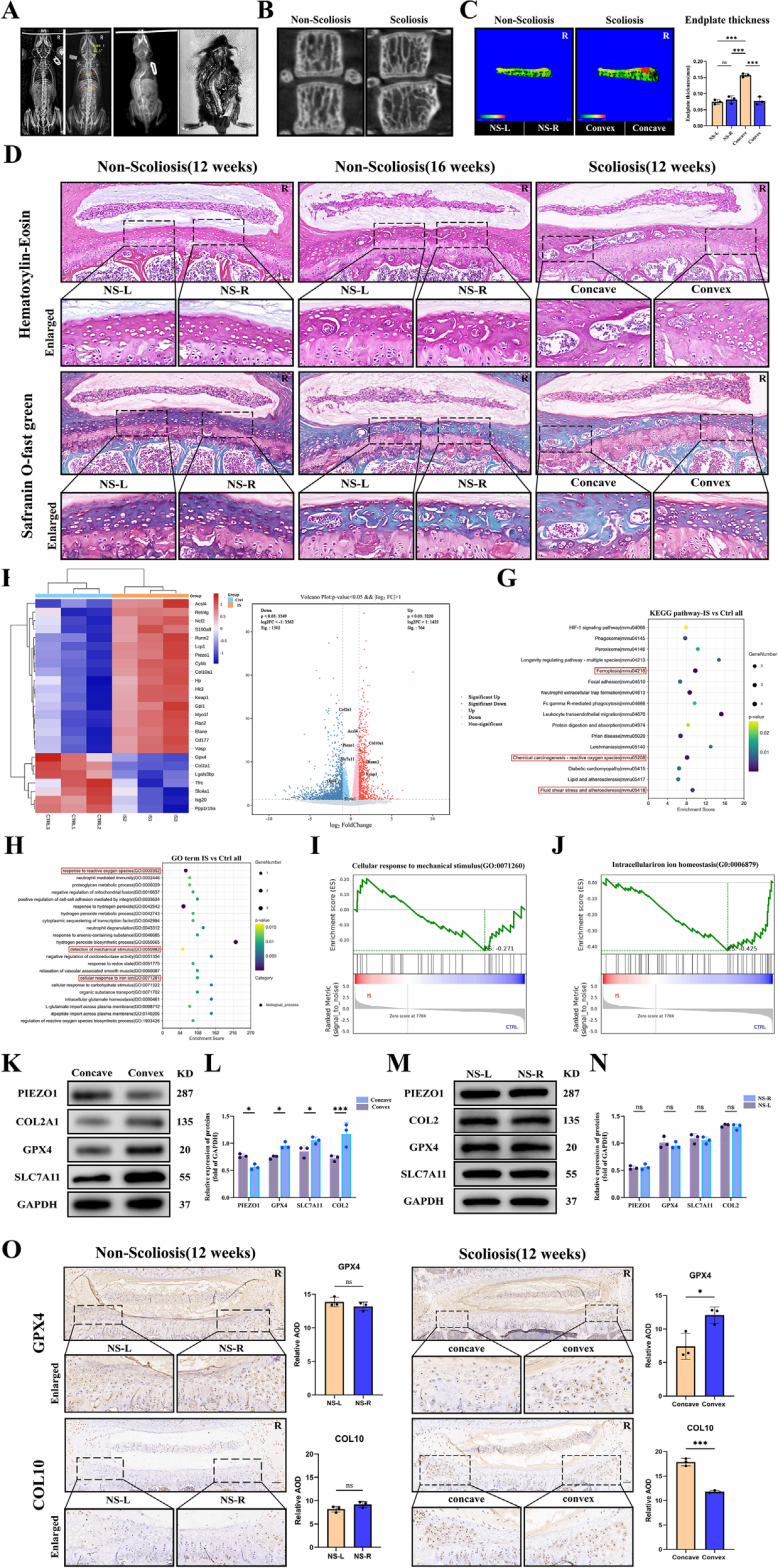
Higher Degree of Ferroptosis and Cartilage Degeneration and Ossification in the Concave Vertebral Growth Plate of Scoliosis. A) Radiological and anatomical verification of scoliosis modeling in mice. B) CT scan comparison between scoliosis and control groups. C) Measurement of the thickness of the vertebral growth plate in the lesion area of scoliosis mice. D) Safranin O‐Fast Green staining and Hematoxylin and Eosin (HE) staining of growth plate cartilage in 12‐week‐old and 16‐week‐old mice without scoliosis and 12‐week‐old mice with scoliosis. E) Heatmap of gene expression in the cartilage of the concave vertebral growth plate near the lesion area of scoliosis mice compared with the control group. F) Volcano plot of differential genes in the cartilage of the concave vertebral growth plate near the lesion area of scoliosis mice compared with the control group. G,H) KEGG and GO bubble plots of the cartilage of the concave vertebral growth plate near the lesion area of scoliosis mice compared with the control group. I,J) GSEA analysis reveals changes in response to mechanical stimulus and regulation of iron metabolic pathways. K–N) Western blot analysis of PIEZO1, cartilage matrix markers (COL2), and ferroptosis markers (GPX4, SCL7A11) in the growth plate cartilage of the vertebrae near the concave side and the convex side of the scoliotic mice, and the corresponding positions in the nonscoliotic controls. O) IHC of GPX4 and COL10 in the vertebral growth plates of 12‐week‐old mice without scoliosis and 12‐week‐old mice with scoliosis. All statistical data are presented as mean ± SD, *n* ≥ 3. ns (not significant), *P < 0.05, **P < 0.01, ***P < 0.001.Statistical analyses were performed using GraphPad Prism software, with one‐way ANOVA followed by Dunnett's post hoc tests and the independent‐sample *t*‐test.

Our previous research affirmed the disparity in PIEZO1 expression between the convex and concave sides of the vertebral growth plate in scoliosis.^[^ ^12^ ^]^ The scoliosis mouse model was constructed by truncating the upper limbs and tails of C57/B6J mice via the aforementioned approach, and the success of the scoliosis mouse model was verified by X‐ray and anatomy (**Figure** **2**A). To explore the dissimilarity in endplate ossification between the convex and concave sides, micro‐CT was carried out on the apical vertebrae area of the lateral curvature, and mineral density analysis was executed. Compared to convex regions, concave endplates exhibited more pronounced ossification characterized by narrowed disc spaces, osteophyte formation, and increased endplate thickness (Figure 2B,C). Histological assessment via Safranin O‐Fast Green and H&E staining of vertebral tissues from 12‐week‐old nonscoliotic mice, 16‐week‐old nonscoliotic mice, and 12‐week‐old scoliotic mice demonstrated that physiological growth plate transformation into bony endplates progresses symmetrically in healthy individuals. This process manifests as gradual replacement of red‐stained cartilage by green collagenous bone matrix, culminating in trabecular bone formation within the growth plate over time. In scoliotic mice, however, asymmetric ossification was observed between concave and convex sides. Premature ossification occurred in concave regions, where 12‐week‐old scoliotic mice exhibited ossification patterns equivalent to 16‐week‐old controls (Figure 2D).

The apical vertebra, defined as the most rotated and displaced segment at the apex of the scoliotic curve,^[^ ^27^, ^28^ ^]^ was identified in scoliotic mice. To meet transcriptome sequencing requirements for RNA integrity and quantity, concave‐side vertebral growth plate cartilage from apical vertebrae in the scoliosis group and ipsilateral growth plate cartilage from age‐matched nonscoliotic control mice (same batch) were harvested for RNA sequencing analysis. The microarray outcomes demonstrated that the expression of PIEZO1 escalated on the concave side. Additionally, the expression of PIEZO1 was positively correlated with the expression of the ferroptosis‐related gene (ACSL4), and negatively correlated with GPX4 and SCL7A11. GO, KEGG, WikiPathway, and Reactom enrichment analysis and GSEA indicated that the stress on the concave side of scoliosis influenced the mechanosensation and ferroptosis‐related pathways (Figure 2E–J; Figure S1A–D, Supporting Information).

Western blot analysis corroborated these findings. Vertebral growth plate cartilage from concave and convex sides of scoliotic mice was dissected, pooled separately, and subjected to total protein extraction. Corresponding tissues from nonscoliotic controls underwent identical processing. Comparative analysis revealed differential expression between concave and convex regions in scoliosis: PIEZO1 expression varied significantly, accompanied by disparities in chondrogenic matrix protein COL2 and ferroptosis markers GPX4, SLC7A11 (Figure 2K,L). Control tissues exhibited no laterality‐dependent differences in these markers (Figure 2M,N). The IHC further demonstrated elevated positivity for COL10 (a chondrocyte degeneration marker) and reduced GPX4 staining in concave versus convex regions of scoliotic mice – differential expression patterns absent in controls (Figure 2O). Collectively, these data suggest heightened ferroptosis and accelerated endplate ossification in concave growth plates, likely associated with upregulated PIEZO1 expression.

### 3.3 Upregulation of PIEZO1 in Vertebral Growth Plate Chondrocytes and Enhanced Ferroptosis Accelerating Degeneration and Ossification of Vertebral Growth Plate Chondrocytes Due to Enhanced Mechanical Stress

**Figure 3:**
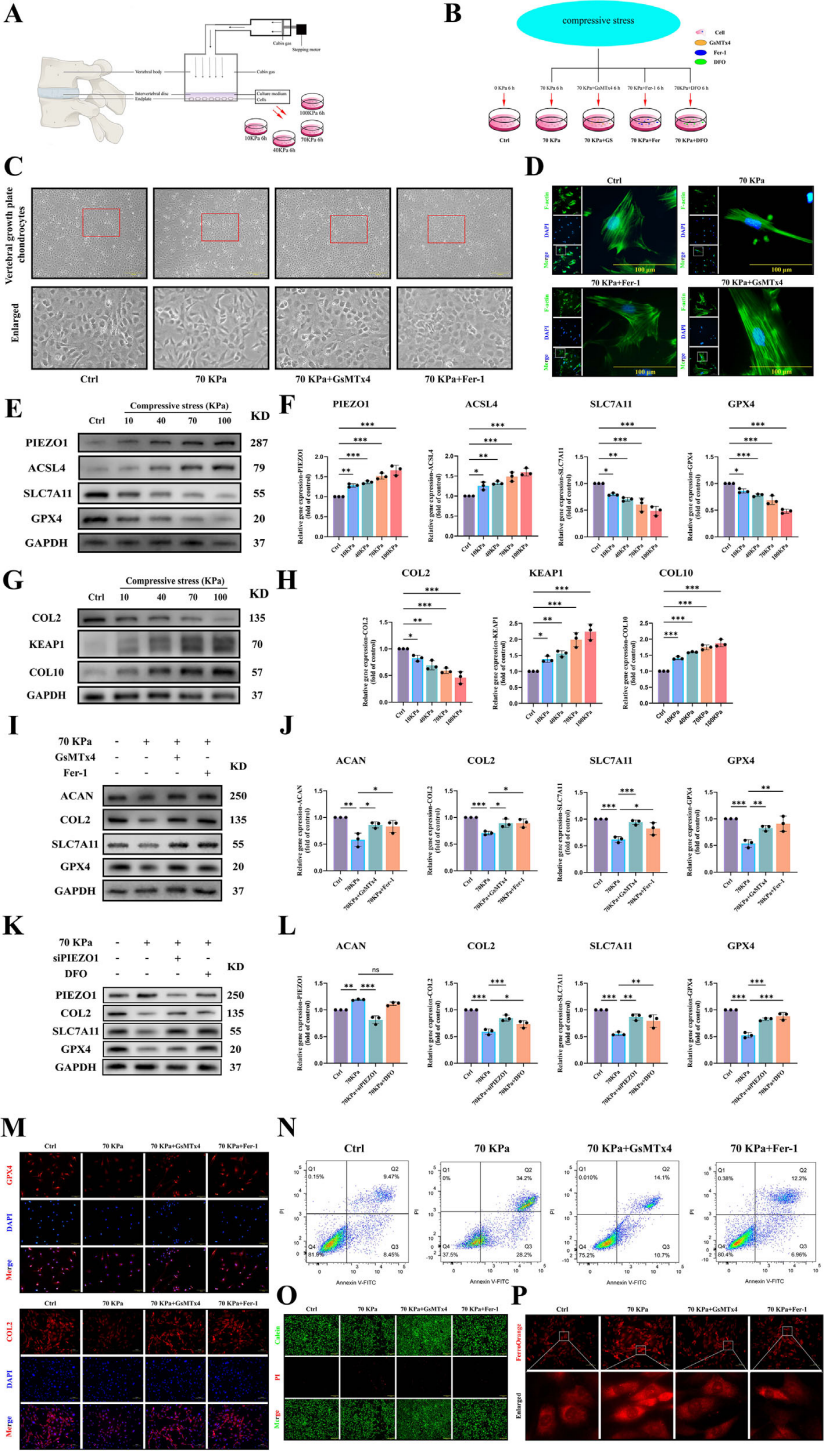
Upregulation of PIEZO1 in Vertebral Growth Plate Chondrocytes and Enhanced Ferroptosis Accelerating Degeneration and Ossification of Vertebral Growth Plate Chondrocytes Due to Enhanced Mechanical Stress. A) Schematic diagram of the mechanical stimulation device. B) Schematic representation of cells treated with different drugs under 70 KPa mechanical stress. C) Morphology of vertebral growth plate chondrocytes from mice treated with GsMTx4 or Fer‐1 under 70 KPa mechanical stimulation for 6 h d^−1^ over 3 d. D) Observation of the cytoskeleton using phalloidin. E–H) Western blot and RT‐PCR analyses of iron metabolism markers (ACSL4, SLC7A11, and GPX4), chondrogenesis‐related markers (ACAN, COL2, COL10), and oxidative damage marker (KEAP1) after mechanical stimulation of different intensities and treatment with GsMTx4 or Fer‐1 under 70 KPa for 6 h d^−1^ over 3 d. I–L) Western blot and RT‐PCR analyses of iron metabolism marker (GPX4, SLC7A11) and chondrogenesis‐related marker (ACAN, COL2) after treatment with GsMTx4 or Fer‐1 and siPIEZO1 or DFO under 70 KPa mechanical stimulation for 6 h d^−1^ over 3 d. M) Immunofluorescence staining of iron metabolism marker (GPX4) and chondrogenesis‐related marker (COL2) after treatment with GsMTx4 or Fer‐1 under 70 KPa mechanical stimulation for 6 h d^−1^ over 3 d. N–P) Flow cytometry apoptosis detection, live/dead cell staining, and ferrous ion staining (Ferrous Orange staining) after treatment with GsMTx4 or Fer‐1 following mechanical stimulation at 70 KPa for 6 h per day over 3 d. All statistical data are presented as mean ± SD, *n* ≥ 3. ns (not significant), *P < 0.05, **P < 0.01, ***P < 0.001. Statistical analyses were performed using GraphPad Prism software, with one‐way ANOVA followed by Dunnett's post hoc tests and the independent‐sample *t*‐test.

When scoliosis aggravates, the compressive stress on the concave side intensifies. To elucidate the relationship between PIEZO1 and the degeneration, mineralization, and ferroptosis of vertebral growth plate chondrocytes when the pressure load on the concave side escalates, mouse vertebral growth plate chondrocytes were isolated and cultivated in an iron‐containing medium established using ferric ammonium citrate (FAC). GsMTx4 is currently the only known inhibitor that specifically targets cationic mechanosensitive channels and has been proven to be applicable in the inhibition of PIEZO1.^[^ ^29^, ^30^ ^]^ Ferrostatin‐1 (Fer‐1), a specific ferroptosis inhibitor, scavenges alkoxyl radicals derived from lipid peroxidation.^[^ ^31^ ^]^ Deferoxamine (DFO) functions as an iron chelator that binds free iron ions to form stable complexes, facilitating their excretion and preventing iron overload.^[^ ^32^ ^]^ Vertebral growth plate chondrocytes were subjected to 70 kPa static compressive stress for 6 h daily over 3 d, with or without GsMTx4, Fer‐1, and DFO cotreatment, followed by comprehensive parameter assessment (**Figure** **3**A,B). A custom‐designed static compression device (Patent No: CN202221667470.X) was employed for mechanical loading. To validate loading reliability, PIEZO1 expression under this device was compared against the commercial Flexcell FX‐5000 system at identical loading conditions (70 kPa, 6 h). Both systems comparably upregulated PIEZO1 expression without statistical difference (Figure S1E, Supporting Information).

Bright‐field microscopy and phalloidin fluorescence staining revealed that mechanical stress altered the cell morphology, and GsMTx4 or Fer‐1 mitigated this change (Figure 3C,D). RT‐PCR and western blot results demonstrated that the increase in mechanical stress elevated the expression of PIEZO1 in cells, while the expression of ferroptosis‐related proteins GPX4 and SLC7A11 decreased, the expression of ACSL4 increased, the expression of oxidation damage‐related protein KEAP1 increased, and the expression of cartilage proliferation‐related proteins COL2 and ACNA decreased, and the expression of cartilage degeneration‐related protein COL10 increased(Figure 3E–J; Figure S2A,B, Supporting Information). Prior studies have demonstrated that GsMTx4, a spider venom peptide, inhibits cation mechanosensitive channels, including PIEZO1, but is not a PIEZO1‐specific inhibitor. Concurrently, it has been reported that PIEZO1 can promote iron influx leading to iron overload. Therefore, we employed siRNA (**Table** **4**) to knockdown PIEZO1 expression and added an iron chelator (DFO) subgroup to observe the above parameters. The results showed that siRNA successfully suppressed PIEZO1 expression, while DFO treatment had no effect on PIEZO1 levels. Both siPIEZO1 and DFO ameliorated the expression of COL2, GPX4, and SLC7A11 induced by mechanical stress (Figure 3K,L). PIEZO1 is in an opposite trend to cartilage proliferation (ACAN, COL2) and ferroptosis‐related proteins (GPX4, SLC7A11), and similar outcomes were obtained by immunofluorescence staining (Figure 3M). Orange iron and live/dead cell staining and flow cytometry results indicated that mechanical stress simultaneously augmented the intracellular Fe^2+^ level and cell mortality rate (Figure 3N–P; Figure S2C,D, Supporting Information). The application of GsMTx4 or Fer‐1 attenuated these effects.

**Table 4:** siPIEZO1 sequence.

Target	siPIEZO1 sequence,5′‐3′
PIEZO1	GCCUCGUGGUCUACAAGAU

These phenomena are in accordance with the results of the aforementioned clinical data and animal experiments, suggesting that PIEZO1 may be associated with ferroptosis in vertebral growth plate chondrocytes and mediate the degeneration and ossification of vertebral growth plate chondrocytes through this pathway.

### 3.4 PIEZO1 Overload Accelerates Degeneration and Ossification of Vertebral Growth Plate Chondrocytes by Mediating Ferroptosis and Oxidative Stress

**Figure 4:**
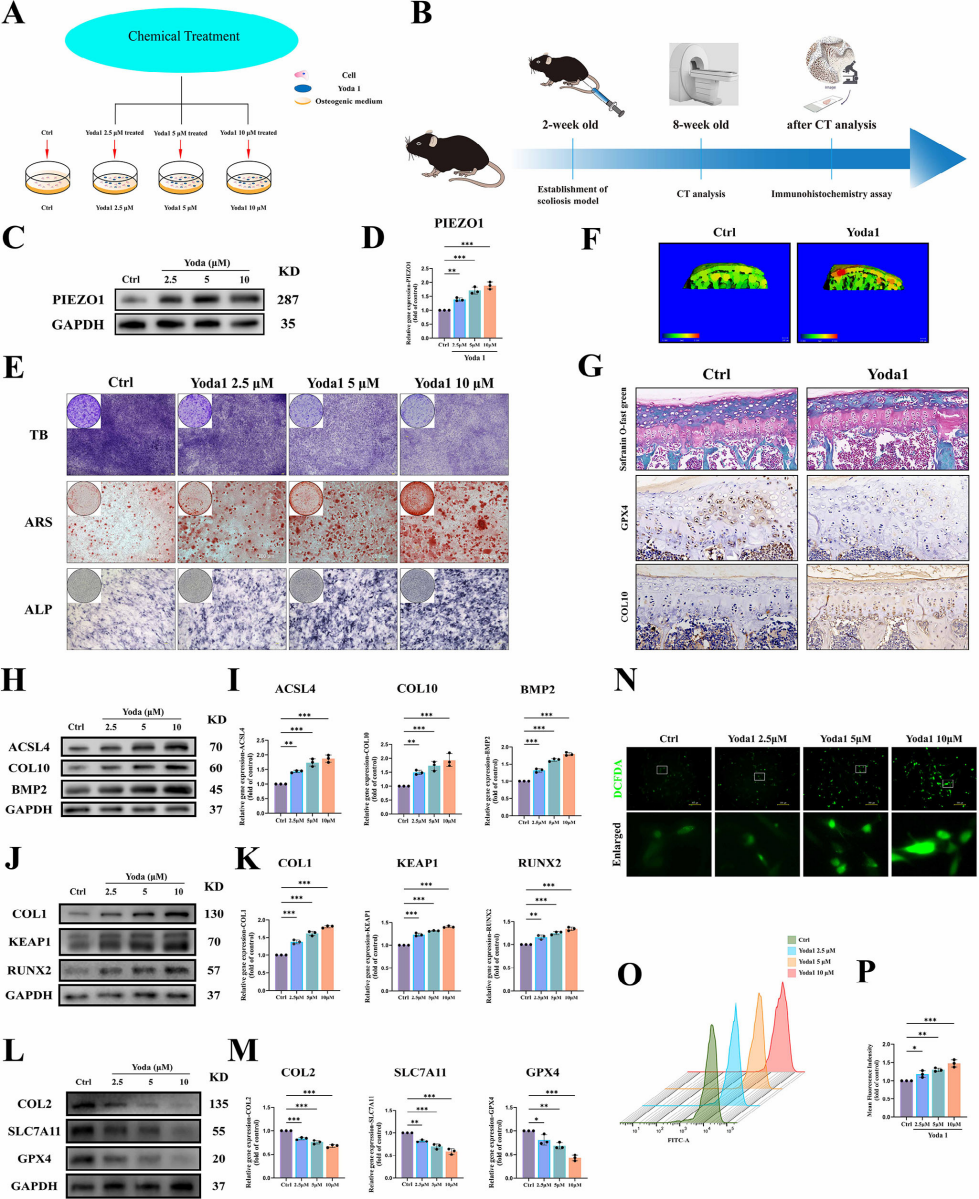
PIEZO1 Overload Accelerates Degeneration and Ossification of Vertebral Growth Plate Chondrocytes by Mediating Ferroptosis and Oxidative Stress. A) A schematic diagram of cells treated with different drugs. B) An experimental schematic diagram of the Yoda1 agonist mouse model. C,D) Western blot and RT‐PCR analyses of PIEZO1 expression under graded concentrations of Yoda1 treatment. (E) Macroscopic and microscopic observations of ARS, ALP, and TB staining under treatment with different concentrations of Yoda1. F) Micro‐CT reconstruction of mice in the Yoda 1 (5 µmol kg^−1^) intraperitoneal injection group and the control group. G) Safranin O‐Fast Green staining and IHC of mice in the Yoda 1 (5 µmol kg^−1^) intraperitoneal injection group and the control group. H–M) Western blot and RT‐PCR analyses of ferroptosis‐related indicators (ACSL4, SLC7A11, GPX4), cartilage degeneration and ossification‐related indicators (COL10, BMP2, RUNX2) and oxidative stress‐related indicator (KEAP1) expression under graded concentrations of Yoda1 treatment. N) Detection of ROS production using the DCFHDA probe under treatment with different concentrations of Yoda1. O,P) Detection and quantitative analysis of ROS production by flow cytometry under treatment with different concentrations of Yoda1. All statistical data are presented as mean ± SD, *n* ≥ 3. ns (not significant), *P < 0.05, **P < 0.01, ***P < 0.001. Statistical analyses were performed using GraphPad Prism software, with one‐way ANOVA followed by Dunnett's post hoc tests and the independent‐sample *t*‐test.

To evaluate the impact of PIEZO1‐mediated ferroptosis on the fate of vertebral growth plate chondrocytes, in vitro and in vivo experiments were carried out. Yoda1, a potent and selective PIEZO1 agonist.^[^ ^33^ ^]^ Vertebral growth plate chondrocytes were cultured in media containing increasing concentrations of Yoda1, followed by subsequent assays (**Figure** **4**A). In C57B/6J mice, starting from 4 weeks of age, Yoda1 (5 µmol kg^−1^) was intraperitoneally injected twice a week for 8 weeks, and the mice were sacrificed at 12 weeks of age for sample collection and detection (Figure 4B). As the initial step, we validated the regulatory effect of Yoda1 on PIEZO1 in vertebral growth plate chondrocytes. Western blot and RT‐PCR analyses demonstrated that PIEZO1 expression increased dose‐dependently with escalating concentrations of Yoda1 (Figure 4C,D). After being treated with different concentrations (0, 2.5, 5, 10 × 10^−6^ m) of Yoda1, alizarin red staining and ALP activity analysis were employed to examine mineralized deposits, and toluidine blue staining was utilized to observe cartilage formation ability. Yoda1 resulted in a dose‐dependent increase in calcium deposition and ALP activity in endplate chondrocytes and the inhibition of cartilage formation (Figure 4E). Similar results were also observed in the in vivo experiments. Three‐dimensional reconstruction micro‐CT images revealed that compared with the control group, a more pronounced increase in endplate thickness was found in the Yoda1 (5 µmol kg^−1^, 4 mL kg^−1^ day^−1^) intraperitoneal injection group. HE and Safranin O‐Fast Green staining demonstrated that the intraperitoneal injection of Yoda1 accelerated the ossification of the growth plate cartilage endplate (Figure 4F). Simultaneously, IHC indicated that compared with the control group, the Yoda1 intraperitoneal injection group exhibited higher COL10 and lower COL2 expression (Figure 4G). Western blot and RT‐PCR analyses demonstrated that Yoda1‐induced PIEZO1 upregulation promoted expression of chondrocyte degeneration‐associated factor COL10, osteogenesis‐related transcription factors (RUNX2, COL1, and BMP2), and oxidative stress‐linked regulator KEAP1, whereas it suppressed expression of cartilage matrix‐related factor COL2. Furthermore, ferroptosis markers exhibited dose‐dependent alterations: GPX4 and SLC7A11 were downregulated while ACSL4 was upregulated (Figure 4H–M; Figure S2E,F, Supporting Information). Based on the above results, it is contended that PIEZO1 may accelerate the ossification of the vertebral growth plate cartilage endplate by promoting ferroptosis.

Ferroptosis is an iron‐dependent nonapoptotic cell death modality characterized by the accumulation of lipid reactive oxygen species (ROS).^[^ ^34^ ^]^ Ferroptosis is intimately associated with oxidative stress.^[^ ^35^, ^36^ ^]^ Oxidative stress plays a crucial role in endplate mineralization and degeneration.^[^ ^37^ ^]^ To investigate the mechanism through which PIEZO1‐mediated ferroptosis expedites the degeneration and ossification of vertebral growth plate chondrocytes, we employed the DCFHDA probe and flow cytometry to assess the production of ROS, and utilized the corresponding kits to evaluate the levels of GSH and MDA. The outcomes demonstrated that an increase in Yoda1 concentration can facilitate the generation of ROS, decrease the level of GSH, and augment the level of MDA (Figure 4N‐P; Figure S2G,H, Supporting Information), suggesting that PIEZO1 can intensify the occurrence of oxidative stress. The results of Western blotting and RT‐PCR analyses can also substantiate this conclusion. The expression of the oxidative stress‐related factor KEAP1 escalates with the rise in Yoda1 concentration (Figure 4J,K; Figure S2F, Supporting Information). The aforementioned evidence indicates that oxidative stress is a key link in PIEZO1‐mediated ferroptosis of growth plate chondrocytes.

### 3.5 PIEZO1 Overload Mediates Ferroptosis and Oxidative Stress in Growth Plate Chondrocytes by Interfering with GPX4, and Inhibiting Oxidative Stress and Ferroptosis Can Alleviate Growth Plate Chondrocyte Degeneration and Ossification

**Figure 5:**
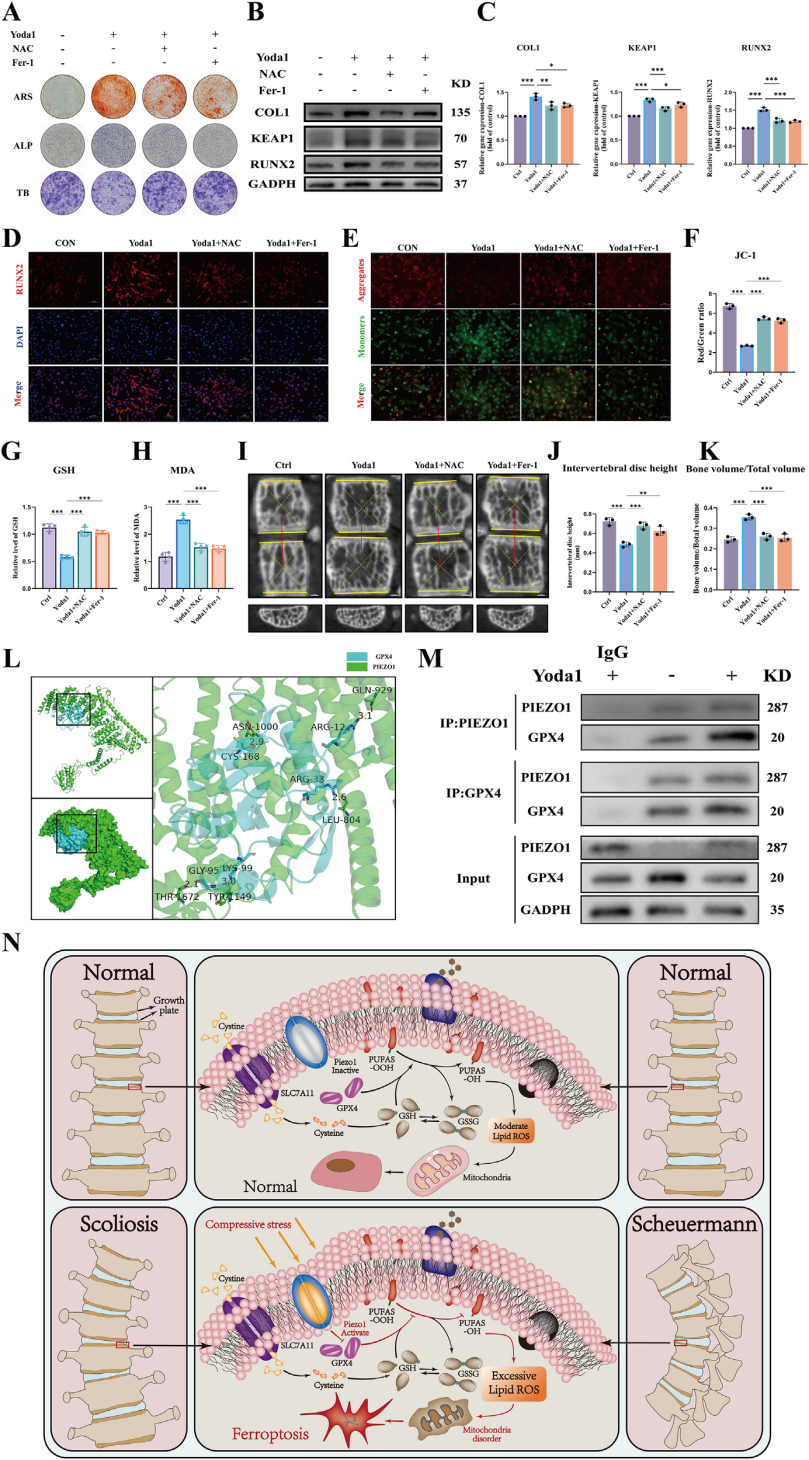
PIEZO1 Overload Mediates Ferroptosis and Oxidative Stress in Growth Plate Chondrocytes by Interfering with GPX4, and Inhibiting Oxidative Stress and Ferroptosis Can Alleviate Growth Plate Chondrocyte Degeneration and Ossification. A) The macroscopic and microscopic observations of ARS, ALP, and TB staining with or without Yoda1, NAC, and Fer‐1. B,C) Western‐blot and PCR analysis of cartilage degeneration and ossification‐related indicators (COL1, RUNX2) and oxidative stress‐related indicator (KEAP1) expression with or without Yoda1, NAC, and Fer‐1. D) Immunofluorescence (IF) of cartilage degeneration and ossification‐related indicator (RUNX2) with or without Yoda1, NAC, and Fer‐1. E,F) JC‐1 assay shows the mitochondrial membrane potential of NPCs. JC‐1 monomers are stained green, and JC‐1 aggregates are stained red, with quantitative analysis of the relative mean fluorescence intensity (MFI). G,H) Detection of glutathione (GSH) and malondialdehyde (MDA). I–K) CT observation and measurement of intervertebral disc space and endplate bone mass, and quantitative analysis of intervertebral disc space height and endplate trabecular bone. L) Hdock molecular docking simulation of PIEZO1 and GPX4. M) Immunoprecipitation (co‐ip) of Piezo1 and GPX4. N) Schematic diagram of the molecular mechanism by which PIEZO1 binds to and interferes with the function of GPX4. All statistical data are presented as mean ± SD, *n* ≥ 3. ns (not significant), *P < 0.05, **P < 0.01, ***P < 0.001. Statistical analyses were performed using GraphPad Prism software, with one‐way ANOVA followed by Dunnett's post hoc tests and the independent‐sample *t*‐test.

We further probed into the impact of inhibiting oxidative stress and ferroptosis on the degeneration and ossification of endplate chondrocytes triggered by PIEZO1 overload. Vertebral growth plate chondrocytes were treated with 10 × 10^−6^ m Yoda 1, 100 × 10^−6^ m antioxidant NAC, and 1 × 10^−6^ m ferroptosis inhibitor Fer‐1. The outcomes of ARS, ALP, and TB staining indicated that 10 × 10^−6^ m Yoda 1 facilitated chondrocyte degeneration and ossification (**Figure** **5**A). Western blot and RT‐PCR analyses revealed enhanced expression of osteogenesis‐related markers (RUNX2, COL1, BMP2) and oxidative stress regulator KEAP1, whereas cartilage matrix markers ACAN and COL2 exhibited suppressed expression levels. Both *N*‐acetylcysteine (NAC) and ferrostatin‐1 (Fer‐1) treatments alleviated these alterations (Figure 5B,C; Figure S2I, Supporting Information). The IF of RUNX2 revealed a consistent trend: Yoda1 treatment increased both the number of red puncta and overall fluorescence intensity compared to controls. Both *N*‐acetylcysteine (NAC) and ferrostatin‐1 (Fer‐1) treatments alleviated these changes (Figure 5D). Subsequently, we employed JC‐1 detection to assess mitochondrial function and further evaluate the effect of Fer‐1 on oxidative stress. The results demonstrated that Yoda1 treatment would interfere with mitochondrial function, while NAC and Fer‐1 could sustain mitochondrial function and alleviate oxidative stress (Figure 5E,F). Lipid peroxidation levels were subsequently assessed using commercial glutathione (GSH) and malondialdehyde (MDA) assay kits. Notably, both NAC and Fer‐1 treatments significantly elevated GSH levels while reducing MDA content compared to Yoda1 monotherapy (Figure 5G,H). Significant in vivo observations were also noted. 3D reconstructed micro‐CT images revealed that Yoda1 treatment reduced intervertebral disc height while promoting trabecular hyperplasia and increased endplate bone density compared to controls, suggesting accelerated degeneration due to growth plate degenerative ossification. Both NAC and Fer‐1 treatments attenuated these pathological changes (Figure 5I–K). These findings demonstrate that inhibiting oxidative stress and ferroptosis alleviates PIEZO1‐overload‐induced degeneration and ossification of vertebral growth plate cartilage. Based on these findings, we propose that ferroptosis serves as a critical mechanistic pathway through which PIEZO1 overexpression drives degradation and ossification of growth plate chondrocytes.

To further elucidate the molecular mechanism underlying PIEZO1's role in promoting ferroptosis, we evaluated the potential interaction between PIEZO1 and GPX4 using molecular docking and co‐immunoprecipitation (CO‐IP) techniques. The protein structures of GPX4 (PDB ID: 5L71) and PIEZO1 (PDB ID: 8IMZ) were retrieved from the Protein Data Bank (PDB) database, and the interaction mode between these two proteins was analyzed using Hdock. The docking results revealed a binding energy of −314.26 kcal mol^−1^ for the GPX4‐PIEZO1 complex, indicating a strong interaction. Specifically, ASP‐599, ASN‐595, ARG‐601, GLU‐576, and ASP‐630 of PIEZO1 formed hydrogen bonds with ARG‐438, ASN‐420, GLU‐418, SER‐249, and ARG‐256 of GPX4, respectively, with hydrogen bond lengths are 2.3, 3.4, 3.5, 2.7, 2.9, 3.0, 2.7 Å, respectively. This implies that PIEZO1 and GPX4 possess the capability to form a stable protein–protein complex (Figure 5L). Simultaneously, the CO‐IP indicates that GPX4 is expressed in the proteins adsorbed by the PIEZO1 antibody. Upregulating PIEZO1 can inhibit the function of GPX4 (Figure 5M).

Collectively, these results demonstrate that PIEZO1 overexpression exacerbates vertebral growth plate degeneration (VGPD) by mediating ferroptosis in growth plate chondrocytes via GPX4 binding and functional inhibition, thereby accelerating cartilage degeneration and ossification (Figure 5N). This mechanism may provide an explanation for the abnormal osteophyte formation and increased bone mineral density observed in the concave high‐pressure area of scoliosis, as found in both clinical and animal studies (Figure 1D,E).

### 3.6 Inhibiting the Expression of PIEZO1 after the Occurrence of Scoliosis can Delay its Progression

Our study demonstrates differential PIEZO1 expression across vertebral growth plates in scoliosis, with significantly higher levels detected on the concave side compared to the convex side. This mediates the occurrence of ferroptosis and induces premature degeneration and ossification of the chondrocytes in the concave side of the vertebral growth plate, resulting in an enlarged difference in the growth plates on both sides of the vertebral body, thereby aggravating the development process of scoliosis. The exacerbated scoliosis further heightens the stress on the concave side of the vertebral growth plate, establishing a vicious cycle and further hindering the development of the vertebral growth plate.

To evaluate whether chondrocyte‐specific PIEZO1 gene knockout would attenuate the deterioration process of scoliosis in vivo, we utilized both WT and PIEZO1‐conditional knockout (PIEZO1‐cKO; Col2‐CreERT, Piezo1^flox/flox^) mice for in‐depth analysis (Figure S2J–L, Supporting Information). Mouse models were established at 3 weeks of age, and following confirmation of scoliosis via X‐ray at 6 weeks, the PIEZO1‐cKO group underwent intraperitoneal injection of tamoxifen (75 mg kg^−1^ d^−1^) for 5 consecutive days to induce specific chondrocyte PIEZO1 knockout in the scoliosis mouse model. Concurrently, we also investigated whether intraperitoneal injection of the PIEZO1 inhibitor GsMTx4 could slow down the progression of scoliosis. After X‐ray confirmation of scoliosis in the WT model mice, a subset of these mice were randomly assigned to receive intraperitoneal GsMTx4 injections (1.5 mg kg^−1^ week^−1^) to create a scoliosis mouse model with systemic PIEZO1 inhibition. The remaining WT mice served as the control group.

**Figure 6:**
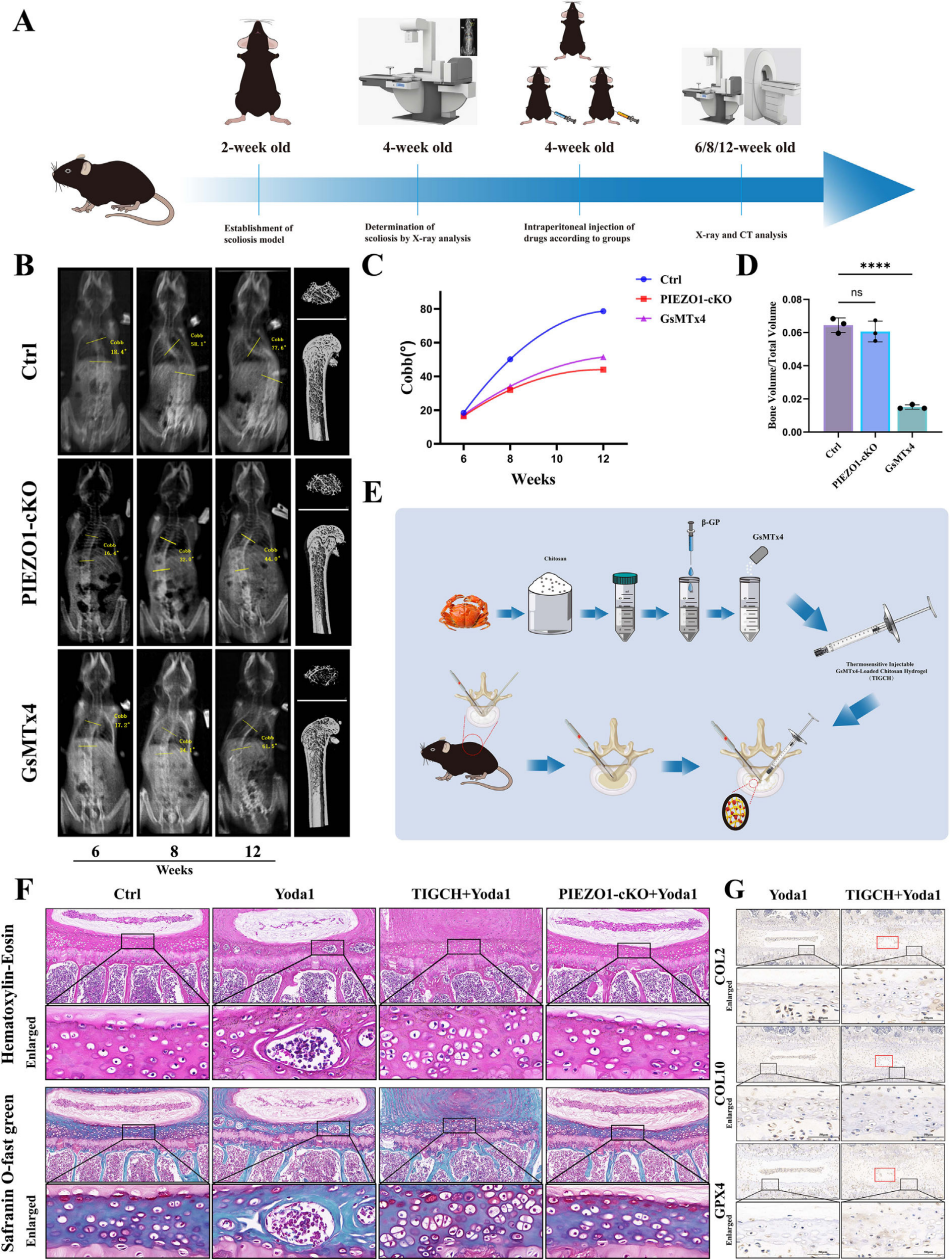
Utilizing PIEZO1 to regulate ferroptosis in vertebral growth plate chondrocytes may be a potential therapeutic mechanism to delay the rapid progression of vertebral growth plate dysplasia diseases. A) Schematic diagram of the experimental design for the treatment of scoliosis model mice with specific chondrocyte PIEZO1 knockout and GsMTx4 injection. B) In vivo assessment of PIEZO1 conditional knockout (cKO) and GsMTx4 therapeutic efficacy in scoliotic mice via whole‐spine radiography and femoral micro‐computed tomography (micro‐CT). C) Line graph recording the progression of Cobb angles in scoliosis mice treated with PIEZO1‐cKO and GsMTx4 application. D) Comparison of femoral trabecular bone in scoliosis mice treated with PIEZO1‐cKO and GsMTx4 at 12 weeks of age. E) Schematic Illustration of Thermosensitive Injectable GsMTx4‐Loaded Chitosan Hydrogel (TIGCH) Fabrication and Microendoscopy‐Assisted In Vivo Administration Workflow. F) HE and Safranin O‐Fast Green staining. G) IHC for COL2, COL10, GPX4 in serial vertebral sections. The red box shows the hydrogel injection site and the cavity caused by the absorption of the residual hydrogel. All statistical data are presented as mean ± SD, *n*≥ 3. ns (not significant), *P < 0.05, **P < 0.01, ***P < 0.001. Statistical analyses were performed using GraphPad Prism software, with one‐way ANOVA followed by Dunnett's post hoc tests and the independent‐sample *t*‐test.

Subsequently, whole‐spine X‐rays were captured at both 8 and 12 weeks of age to monitor the progression of scoliosis. Following this observation, Micro‐CT scans were performed on the femurs of each group. Both specific chondrocyte PIEZO1 knockout and GsMTx4 administration, when compared to the control group, failed to completely arrest the deterioration of scoliosis but did effectively slow down its progression. This outcome validates our research hypothesis (**Figure** **6A–D**).

It is worth observing that, although mice injected with GsMTx4 showed a delayed progression of scoliosis, they also exhibited osteoporosis compared to the control group. In contrast, mice with a specific chondrocyte PIEZO1 knockout did not exhibit this osteoporosis (Figure 6B). This finding indicates that inhibiting PIEZO1 expression after the onset of scoliosis can slow its progression. However, the systemic use of GsMTx4 as a therapeutic intervention to delay scoliosis progression may carry certain risks, necessitating further research to explore alternative methods of administration.

### 3.7 Local Application of GsMTx4 using Chitosan Hydrogel as a Carrier in Combination with Endoscopic Techniques can Delay the Ossification Process of Vertebral Growth Plate Cartilage

Recently, the local application of chitosan as a carrier in cartilage repair has garnered increasing attention.^[^ ^38^, ^39^ ^]^ Leveraging our previous design of a thermosensitive injectable chitosan hydrogel for drug encapsulation,^[^ ^40^ ^]^ we encapsulated GsMTx4 within chitosan hydrogel to create a thermosensitive injectable GsMTx4‐loaded chitosan hydrogel (TIGCH). This innovation aims to explore the feasibility of locally applying GsMTx4 to the vertebral growth plate to improve scoliosis progression.

TIGCH exists as a milky‐white viscous liquid at room temperature, exhibiting syringe‐injectability (Figure S3A, Supporting Information). Upon exposure to 37 °C, it undergoes phase transition to a gel‐like solid (Figure S3B, Supporting Information). Scanning electron microscopy revealed a porous microstructure within TIGCH, with GsMTx4 particles dispersed uniformly throughout the chitosan hydrogel matrix (Figure S3C, Supporting Information). At physiological temperature (37 °C), TIGCH demonstrated sustained release of GsMTx4 accompanied by gradual hydrogel degradation (Figure S3D,E, Supporting Information). Hoechst 33 342 (a cell‐permeant blue‐fluorescent nuclear stain with minimal cytotoxicity) was employed for live‐cell imaging. Fluorescence microscopy quantification showed no significant difference in viable cell counts between TIGCH‐treated and control groups (Figure S3F, Supporting Information). Collectively, these results confirm TIGCH as a biocompatible injectable biomaterial suitable for biomedical applications.

However, due to the unique challenges posed by scoliosis modeling in C57BL/6J mice, such as narrow intervertebral spaces and altered anatomical positions of vertebrae during scoliosis progression, in vivo manipulation is challenging. Consequently, we employed microendoscopic techniques to remove intervertebral discs and implant hydrogel into the intervertebral spaces of 6‐week‐old healthy C57BL/6J mice (Figure 6E; Figure S3G, Supporting Information). Additionally, 6‐week‐old PIEZO1‐conditional knockout (PIEZO1‐cKO) mice were treated with intraperitoneal tamoxifen injections (75 mg kg^−1^ d^−1^) for 5 consecutive days. Following the completion of hydrogel implantation and tamoxifen induction, Yoda 1 (5 µmol kg^−1^) was administered intraperitoneally twice weekly. At 12 weeks of age, the mice were euthanized for histological analysis, including HE staining and Safranin O‐Fast Green staining. The results demonstrated that both TIGCH treatment and chondrocyte‐specific PIEZO1 knockout delayed the Yoda1‐induced ossification of vertebral growth plate cartilage (Figure 6F). Compared with Yoda1‐induced PIEZO1‐overexpressing mice, TIGCH cotreatment significantly increased the proportions of COL2‐ and GPX4‐positive cells while reducing COL10 expression in corresponding intervertebral segments, as evidenced by immunohistochemical staining of cartilage matrix marker COL2, ferroptosis‐inhibiting marker GPX4, and chondrocyte degeneration marker COL10. Voids resulting from absorption of residual hydrogel were observed, while notably greater growth plate cartilage layer thickness was documented in TIGCH‐co‐treated mice (Figure 6G). This suggests that it is theoretically viable to employ chitosan hydrogel as a carrier in combination with endoscopic technology for the local application of GsMTx4 to intervene in vertebral growth plate dysplasia.

## 4 Discussion

The progression of VGPD involves 3D biomechanical alterations of the spine, a phenomenon exclusively observed in humans and select bipedal animals due to unique axial stress loading on the upright vertebral column.^[^ ^4^ ^]^ Biomechanical influences on vertebral growth plates follow the Hueter–Volkmann law, which postulates that compressive stress inhibits vertebral elongation while tensile stress may accelerate it.^[^ ^41^ ^]^ Stokes et al. demonstrated this principle using external fixators to apply compressive (25–75% body weight) or tensile loads to Sprague–Dawley rat caudal vertebrae. Their results confirmed growth suppression under compression and enhanced growth under tension, providing compelling evidence for mechanical regulation of vertebral growth.^[^ ^42^ ^]^ Roaf et al. proposed the “vicious cycle” theory of scoliosis progression: asymmetric spinal curvature generates sustained compressive stress on the concave side while reducing compressive load or generating tensile stress on the convex side due to vertebral deformation. This imbalance inhibits concave‐side growth and accelerates convex‐side growth, exacerbating spinal deformity and causing vertebral wedging.^[^ ^5^ ^]^ Finite element analysis of scoliotic spines further corroborates elevated compressive stress on the concave aspect.^[^ ^43^ ^]^

The mechanosensitive ion channel Piezo1 represents a protein class exhibiting exquisite sensitivity to biomechanical stress signals in vitro. Its discovery offers novel mechanistic insights into bone‐related cellular proliferation, apoptosis, and inflammatory mediator production. Our prior work confirmed PIEZO1 overexpression in concave‐side growth plates of scoliotic spines and its functional impact on chondrocyte apoptosis. Recent studies further reveal PIEZO1's involvement in ferroptosis triggered by iron overload.^[^ ^44^ ^]^

Previous research has established that iron overload exacerbates chondrocyte oxidative stress and cartilage matrix degradation, ultimately contributing to cartilage degeneration.^[^ ^22^, ^45^ ^]^ Moreover, iron overload facilitates the generation of reactive oxygen species, triggering ferroptosis in cells.^[^ ^46^ ^]^ In our study, patients with adolescent idiopathic scoliosis (AIS) were categorized into two groups: those with severe scoliosis (Cobb ≥ 90°) and those with nonsevere scoliosis (Cobb < 90°). Statistical analysis revealed significant differences in serum ferritin levels between the two groups. Radiographic assessments further demonstrated abnormal ossification and cartilage degeneration in the vertebral growth plates on the concave side of AIS patients, hinting at the presence of iron overload in severe scoliosis cases. These observations suggest that ferroptosis in vertebral growth plate chondrocytes may play a pivotal role in AIS progression. However, current evidence remains indirect, necessitating more direct validation. Given AIS predominantly affects adolescents and standard corrective surgery rarely involves growth plate resection, procuring human vertebral growth plate specimens proves exceptionally challenging. Partial samples become accessible only during hemivertebra excision procedures.

We obtained paired concave/convex growth plate tissues from one AIS patient undergoing hemivertebra resection. Prussian blue staining revealed significantly deeper iron deposition on the concave side, corroborating iron overload. Ferroptosis characteristically features mitochondrial shrinkage with reduced or absent cristae. Such mitochondrial dysfunction promotes reactive oxygen species (ROS) generation, exacerbating oxidative stress and lipid peroxidation. Transmission electron microscopy (TEM) of extracted chondrocytes demonstrated these diagnostic mitochondrial alterations specifically in concave‐side tissues, providing direct evidence of ferroptosis. Concurrent immunohistochemistry further confirmed elevated oxidative stress marker KEAP1, downregulated ferroptosis‐related protein GPX4, and increased PIEZO1 expression in concave versus convex growth plates.

We successfully established a scoliosis model in C57BL/6J mice by adopting the modeling methodology outlined by Machida et al.^[^ ^6^, ^47^, ^48^ ^]^ Consistent with our hypothesis, radiographic evaluation combined with safranin O/fast green and H&E staining revealed marked degeneration and ossification in the growth plates of scoliotic mice. Transcriptomic sequencing of concave‐side growth plate chondrocytes versus ipsilateral control tissues identified significant differential expression in PIEZO1, ferroptosis‐related, and cartilage degeneration/ossification‐associated genes. Immunohistochemical (IHC) analysis further demonstrated increased numbers of COL10‐positive chondrocytes and elevated levels of the ferroptosis‐inhibiting protein GPX4 in concave endplates compared to convex counterparts. These findings were consistently replicated in vitro.

Ferroptosis constitutes a regulated cell death modality propelled by iron‐dependent lipid peroxidation, particularly targeting phospholipids enriched with polyunsaturated fatty acids (PL‐PUFAs) within the plasma membrane and organelle membranes.^[^ ^34^ ^]^ The induction of ferroptosis hinges on the peroxidation of PL‐PUFAs, with acyl‐CoA synthetase long‐chain family member 4 (ACSL4) playing a pivotal role in catalyzing the activation and membrane incorporation of PUFAs, thereby dictating the sensitivity to ferroptosis.^[^ ^49^, ^50^, ^51^ ^]^ Glutathione peroxidase 4 (GPX4) and solute carrier family 7 member 11 (SLC7A11) serve as crucial regulators of ferroptosis. Specifically, GPX4 employs glutathione (GSH) to neutralize lipid peroxidation products,^[^ ^14^ ^]^ whereas SLC7A11 facilitates the import of cystine for GSH synthesis.^[^ ^15^ ^]^ Our study reveals that ferroptosis, induced by either elevated compressive stress or PIEZO1 activation, is marked by an upregulation of ACSL4 expression coupled with a downregulation of GPX4 and SLC7A11 expression.

Chondrocytes constitute the exclusive cellular component of the vertebral growth plate in the spine. PIEZO1 is a mechanosensitive ion channel highly expressed within vertebral growth plate cartilage.^[^ ^12^ ^]^ Furthermore, existing research supports the involvement of PIEZO1 signaling in ferroptosis, a form of cell death induced by iron overload, through the inhibition of the BMP‐SMAD pathway, ultimately facilitating iron accumulation and ferroptosis.^[^ ^52^, ^53^, ^54^ ^]^ Notably, E756del, a mild gain‐of‐function mutation in PIEZO1 prevalent in one‐third of individuals of African descent, serves as a robust indicator of elevated plasma iron levels.^[^ ^53^, ^55^ ^]^ Studies have indicated that augmented PIEZO1 expression may exacerbate chondrocyte damage and matrix degradation, leading to cartilage degeneration and, consequently, the advancement of osteoarthritis.^[^ ^56^, ^57^ ^]^

In our study, we explored whether PIEZO1 contributes to vertebral growth plate cartilage degeneration, thereby influencing the progression of scoliosis. To this end, we isolated chondrocytes from the vertebral growth plate and subjected them to various intensities of compressive stress or concentrations of Yoda1 to mimic upregulated PIEZO1 expression in vitro. Our findings demonstrated that both compressive stress and Yoda1 treatment augmented the expression of oxidative damage markers (KEAP1), cartilage degeneration markers (COL10), and osteogenic calcification markers (COL1, RUNX2), while concurrently reducing the expression of chondrogenesis markers (COL2, ACAN). These changes facilitated chondrocyte degeneration and calcification. Furthermore, Yoda1 treatment enhanced ALP activity, calcium nodule formation, and cartilage degeneration in vertebral growth plate chondrocytes. Our in vivo experiments corroborated these findings, revealing accelerated ossification of the vertebral growth plate, elevated COL10 expression, and decreased GPX4 expression in mice injected with Yoda1. Based on our collective results, we propose that upregulated PIEZO1 expression induced by Yoda1 and compressive stress treatment accelerates ferroptosis in growth plate chondrocytes, thereby expediting their degeneration and calcification.

Reported studies have demonstrated that PIEZO1 can attenuate the acceleration of GPX4 by regulating Ca^2+^ to intervertebral disc degeneration (IVDD).^[^ ^44^ ^]^ Herein, we also concentrated on the interaction between PIEZO1 and GPX4 in vertebral growth plate chondrocytes. The outcomes of molecular docking simulation and co‐immunoprecipitation both imply that PIEZO1 and GPX4 might bind, which appears to be a novel mode of action. PIEZO1 is a transmembrane protein localized in the cell membrane. GPX4 is freely present in the cytoplasm and is the sole enzyme capable of eliminating lipid peroxides embedded in the cell membrane, which is crucial for cells to withstand lipid peroxidation and cell death. Based on our experimental results, it can be reasonably hypothesized that when compressive stress is excessive, the expression of PIEZO1 is augmented, thereby enhancing the binding with GPX4 that contacts the cell membrane and functions in removing lipid hydrogen peroxide products, and reducing its expression. This leads to a competitive inhibition in the removal of lipid hydrogen peroxide products, thereby causing an upregulation of ferroptosis and ultimately accelerating the degeneration and ossification of the vertebral growth plate cartilage.

This study ascertained the role of PIEZO1 in ferroptosis induced by compression stress overload. The augmented expression of PIEZO1 leads to ferroptosis in vertebral growth plate chondrocytes via oxidative stress, while facilitating the dysplasia of the vertebral growth plate. Antioxidants (NAC) and ferroptosis inhibitors (Fer‐1), as well as the PIEZO1 inhibitor GsMTx4, can effectively suppress the degeneration and ossification of endplate chondrocytes caused by the upregulation of PIEZO1. The subsequent animal experiment results indicated that although the specific knockout of chondrocyte PIEZO1 and the injection of GsMTx4 could not halt the deterioration of scoliosis, they retarded the process of its deterioration to a certain degree.

Surgical correction of severe scoliosis requires extensive incisions with significant tissue trauma, yields inferior corrective outcomes compared to mild cases, and carries substantially higher operative risks.^[^ ^58^, ^59^ ^]^ We are endeavoring to find a method to retard the progression of scoliosis in order to prevent mild scoliosis from transforming into severe scoliosis. As previously stated, the direct in vivo injection of the PIEZO1 inhibitor GsMTx4 can alleviate the deterioration process of scoliosis to a certain extent. However, the observation of a formation disorder in femoral trabecular bone suggests the occurrence of osteoporosis. Existing studies have shown that PIEZO1 has a positive effect on osteoblasts.^[^ ^60^ ^]^ Systemic application of GsMTx4 may inhibit the positive effect of PIEZO1 on osteoblasts, thereby inducing the occurrence of osteoporosis. Consequently, there are certain risks associated with the systemic application of GsMTx4. Conversely, no osteoporosis is observed in the specific knockout of PIEZO1 in chondrocytes, indicating the possibility of local application of GsMTx4 on cartilage. Thus, we employ the scheme of the thermosensitive injectable chitosan hydrogel encapsulating the drug,^[^ ^40^ ^]^ encapsulating GsMTx4 in the chitosan hydrogel to form The Thermosensitive Injectable GsMTx4‐Loaded Chitosan Hydrogel (TIGCH), for the preliminary exploration of local application. The degradation products of chitosan can be absorbed and will not accumulate in the body or generate immunogenicity.^[^ ^38^, ^61^ ^]^ It is extensively utilized in biomedicine and preparations due to its biodegradability, low toxicity, and excellent biocompatibility, and has been applied in cartilage repair.^[^ ^39^, ^62^, ^63^ ^]^ Owing to the small spinal tissue morphology of the scoliosis model mice, local injection of the hydrogel is challenging. We carried out the hydrogel injection with the assistance of a micro‐endoscope and achieved results similar to the direct application. Our application exploration suggests a novel and potential adjunctive treatment plan for scoliosis, namely the combined application of minimally invasive endoscopy and biological drug‐loaded materials.

These results can indicate that PIEZO1 is an efficient regulator of ferroptosis in spinal vertebral growth plate chondrocytes. Under compressive stress overload, PIEZO1 is closely associated with the progression of scoliosis via ferroptosis. This indicates the potential worth of regulating PIEZO1 in new treatment protocols for growth plate dysplasia diseases represented by scoliosis.

## 5 Conclusion

In conclusion, our study demonstrates that PIEZO1 interferes with the anti‐lipid peroxidation function of GPX4, leading to increased intracellular oxidative stress and aggravated ferroptosis, thereby causing vertebral growth plate dysplasia. Employing PIEZO1 to regulate ferroptosis in vertebral growth plate chondrocytes might be a potential therapeutic approach to retard the rapid advancement of vertebral growth plate dysplasia diseases.

## Conflict of Interest

The authors declare no conflict of interest.

## Author Contributions

F.‐C. contributed to the research design, analysis of the data, and manuscript writing. F.S.‐P. and Z.C.‐C. contributed to the acquisition and analysis of the data and wrote the program. S.Q.‐C., Y.Y.‐F., and H.‐Z. contributed to the acquisition and analysis of the data. J.Y.‐L. and G.H.‐G. contributed to the acquisition and analysis of the data. Z.S.‐J. and G.D.‐W. offered and organized the clinical impact data. Y.M.‐X., T.‐L., J.‐D., and X.Z.‐J. initiated and designed the study. All authors were fully involved in the study and approved the final version of this manuscript.

## Supporting information



Supporting Information

## Data Availability

The raw sequence data reported in this paper have been deposited in the Genome Sequence Archive (Genomics, Proteomics & Bioinformatics 2021) in National Genomics Data Center (Nucleic Acids Res 2022), China National Center for Bioinformation/Beijing Institute of Genomics, Chinese Academy of Sciences (GSA: CRA019753) that are publicly accessible at https://ngdc.cncb.ac.cn/gsa. Other data used to support the results of this study can be obtained from the corresponding author upon request.
