# Supplementary material for: PIEZO1‐GPX4 Axis Mediates Mechanical Stress‐Induced Vertebral Growth Plate Dysplasia via Ferroptosis Activation
Source: Adv Sci (Weinh). 2025 Jul 26;12(39):e02052. doi: 10.1002/advs.202502052 (PMC12533149; doi:10.1002/advs.202502052)
Supplement: Supplementary file 1 — Supporting Information [file ADVS-12-e02052-s001.docx]

**Supplementary Information**

**Thermosensitive Hydrogel-Mediated Local Inhibition of the PIEZO1-GPX4 Axis Attenuates Mechanical Stress-Induced Vertebral Growth Plate Dysplasia via Ferroptosis Suppression**

Fei Chen, MD^1,#^, Fushuai Peng, MS^1,#^, Shuqing Chen, MS^1^, Yukun Du, MD^1^, Jianyi Li, MD^1^, Yuanyuan Fan, MS^3^, Zichen Cui, MD^1^, Guanghui Gu, MD^1^, Han Zhang, MS^1^, Zhensong Jiang, MD^2^, Guodong Wang, MD^2^, Xingzhi Jing, MD^2,^*, Jun Dong MD^2,^*, Tao Li, MD^2,^* and Yongming Xi, MD^1,^*

^1^Department of Spinal Surgery, The Affiliated Hospital of Qingdao University, Qingdao, Shandong, 266071, China.

^2^ Department of Spine Surgery, Shandong Provincial Hospital Affiliated to Shandong First Medical University, Jinan, Shandong, 250021, China.

^3^Shandong Public Health Clinical Center, Shandong University, Shandong 250013, China

**This supporting information includes:**

Supplementary Figures S1 to S3

**Figure S1**

**
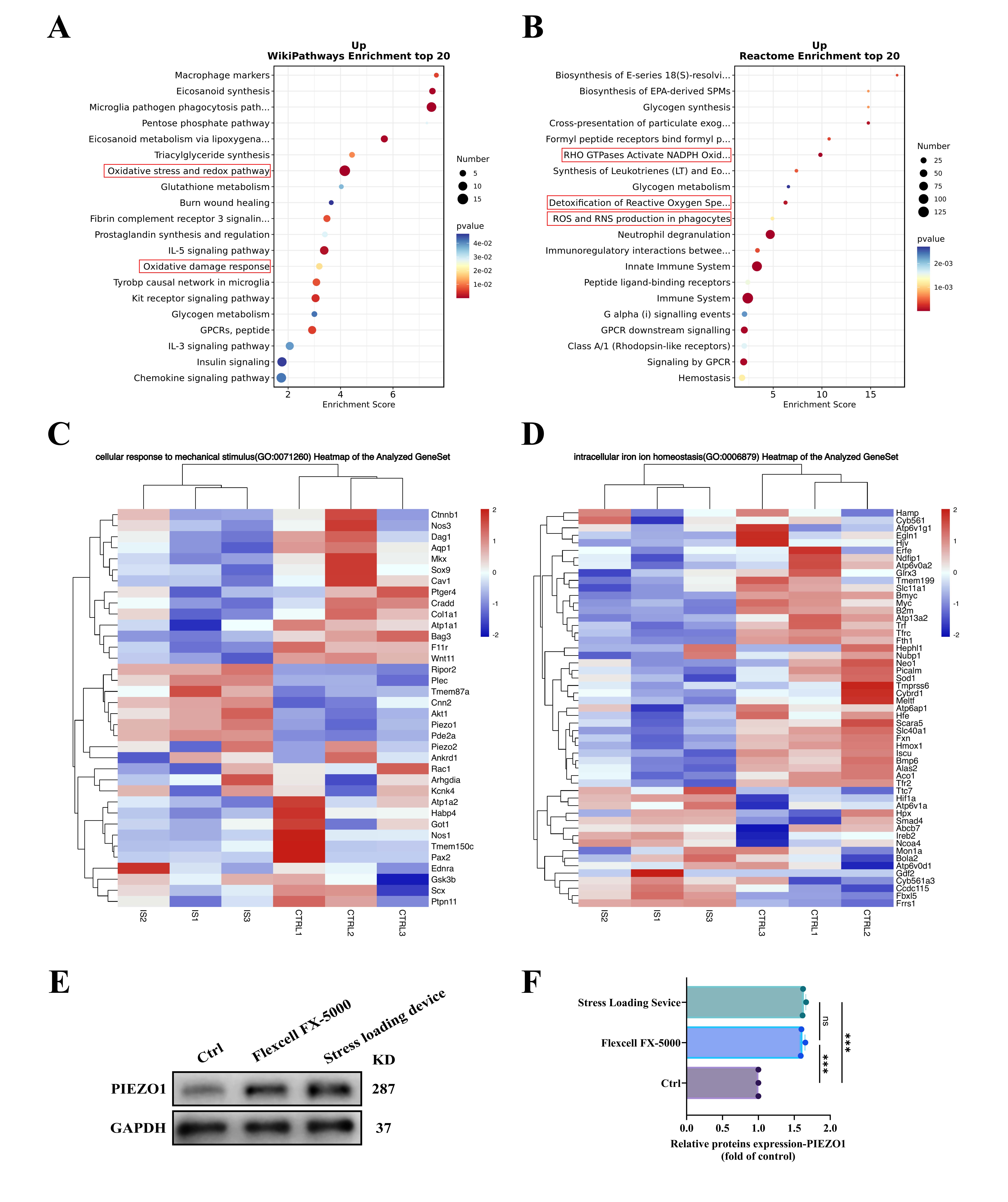
**

**Figure S1 Mechanical stress imposed on the concave side of scoliosis impacts mechanotransduction and pathways associated with ferroptosis.**

**(A, B)** WikiPathway and Reactome bubble plots of the cartilage of the concave vertebral growth plate near the lesion area of scoliosis mice compared with the control group. **(C)** Cellular response to mechanical stimulus(go:0071260) heatmap of the analyzed geneset. **(D)** Intracellular iron ion homeostasis(go:0006879) heatmap of the analyzed geneset. **(E)** Use Western blot to compare the expression of PIEZO1 between the Flexcell FX-5000 pressure loading system and the pressure loading instrument used in the experiment.

**Figure S2.**


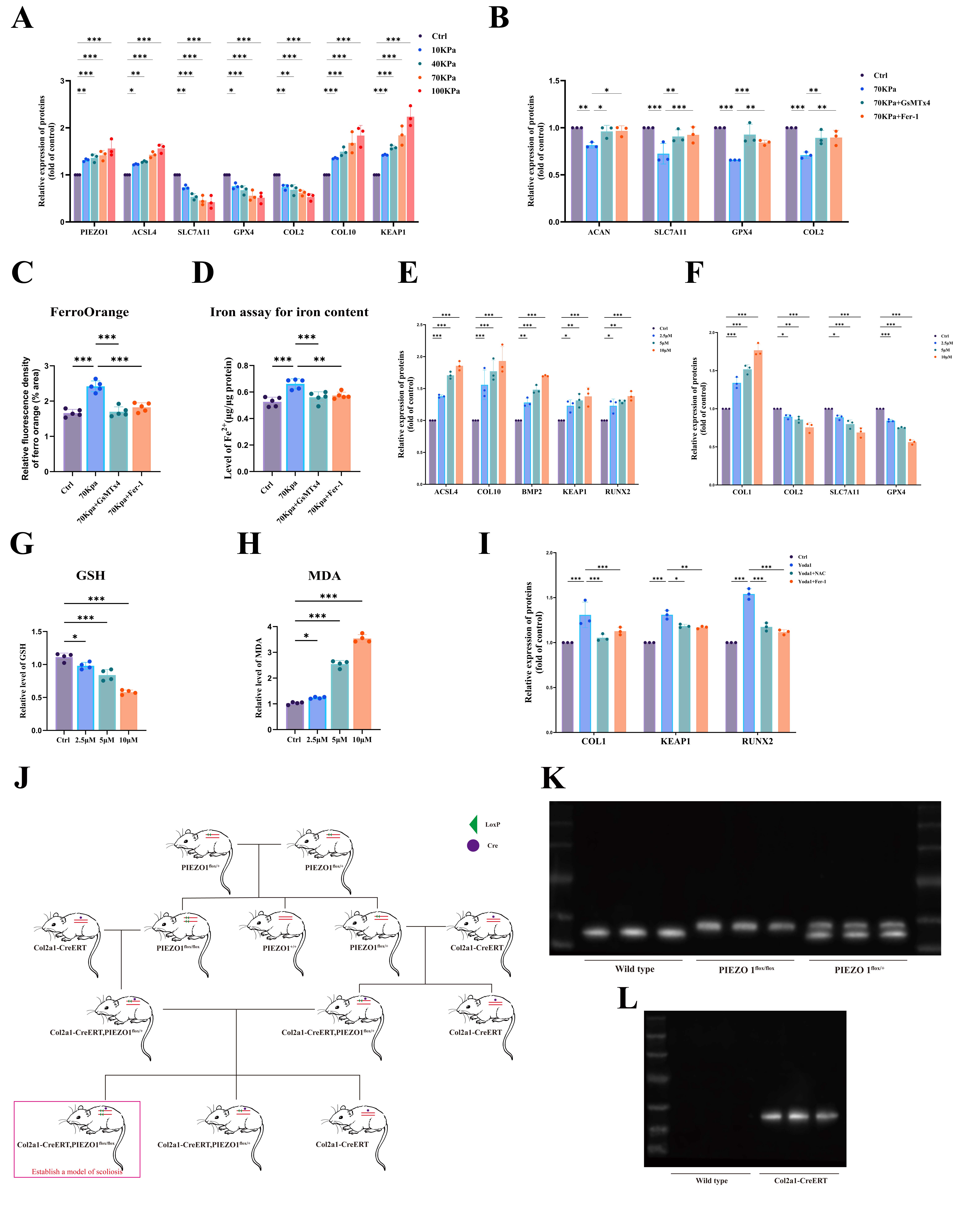


**Figure S2** **Supplementary data after mechanical stimulation of different intensities and different concentrations of Yoda1 treatment, and breeding strategies and gene identification of gene-edited mice.**

**(A)** Quantitative analysis of protein expressions after mechanical stimulation of different intensities. **(B)** Quantitative analysis of protein expressions after treatment with GsMTx4 or Fer-1 under 70 KPa for 6 hours/day over 3 days. (**C and D)** Detection of intracellular Fe^2+^ using FerroOrange and Iron Assay Kit quantitative analysis treatment with GsMTx4 or Fer-1 under 70 KPa for 6 hours/day over 3 days. **(E and F)** Quantitative analysis of protein expressions after different concentrations of Yoda1 treatment. **(G and H)** Utilized the corresponding kits to evaluate the levels of GSH and MDA after different concentrations of Yoda1 treatment. **(I)** Quantitative analysis of treatment with GsMTx4 or Fer-1 under 10μM Yoda1. (**J)** Breeding strategies for Piezo1^flox/flox^, Col2-CreERT (Piezo1-cKO) mice. (**K and L).** Gene identification results: Homozygotes (Piezo1^flox/flox^) :210bp. Heterozygotes (Piezo1^flox/+^) 210/142bp. Wild type (Piezo1^+/+^) 142bp; Col2a1- CreERT: 358bp. Wild type: No stripe.

**Figure S3.**


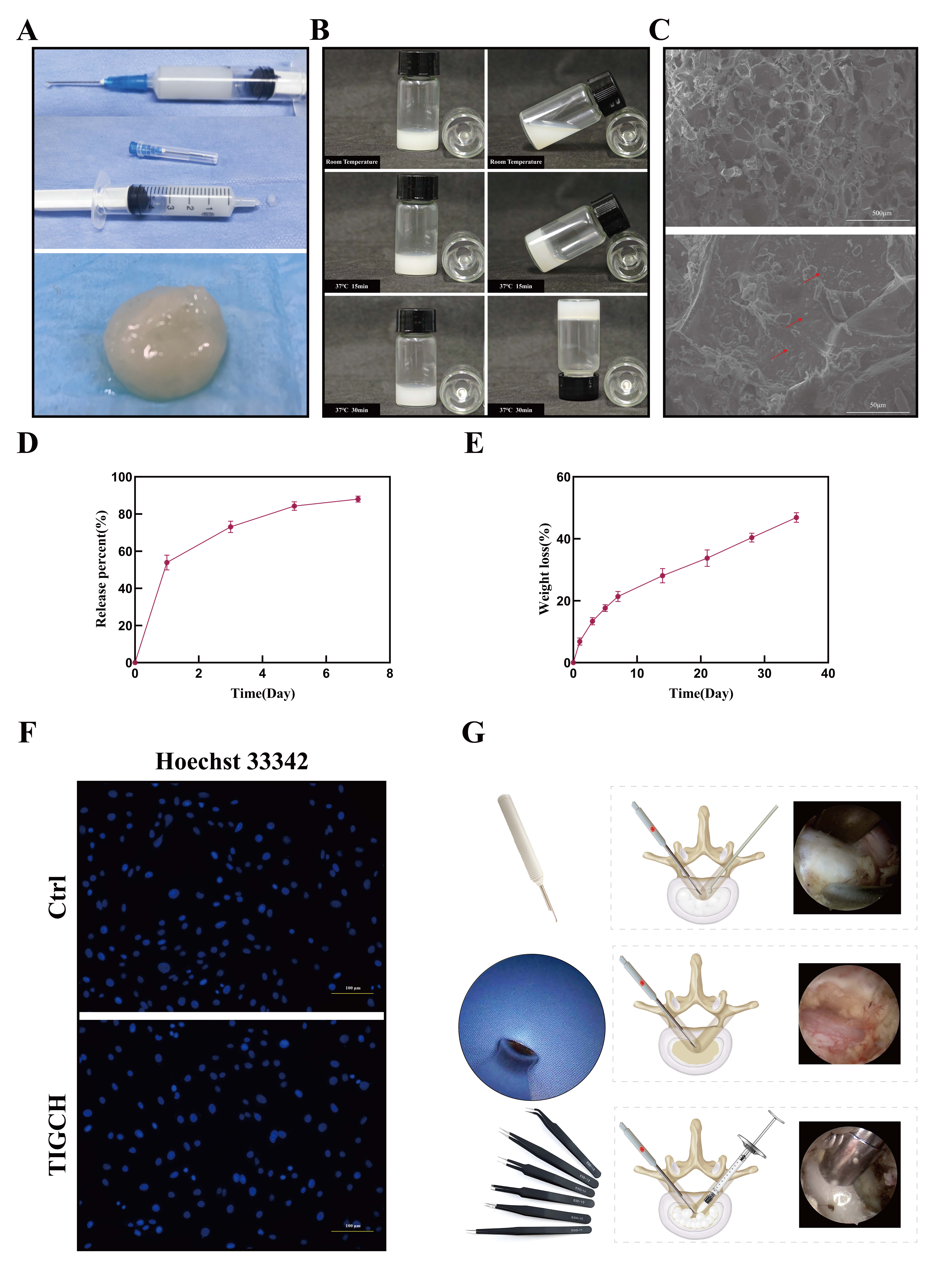


**Figure S3 Locally apply GsMTx4 using chitosan hydrogel as a carrier in combination with endoscopic techniques.**

**(A)** Shape of hydrogel carrier. **(B)** Thermosensitivity test of TIGCH (**C)** Scanning Electron Microscopy of Hydrogel and Encapsulation of GsMTx4 Particles. **(D)** Drug sustained-release test of TIGCH. **(E)** Degradation test of TIGCH. **(F)** Test the biocompatibility of TIGCH using Hoechst 33342. **(G)** Under endoscopic guidance, surgical instruments were employed for nucleus pulposus removal followed by hydrogel injection.
